# Layer-by-Layer Coated Alginate Hydrogels with Antibacterial Activity Based on Bacteriophage and Curcumin Combination

**DOI:** 10.1021/acsomega.5c02562

**Published:** 2025-07-23

**Authors:** Ayse Guren, Aysenur Yucefaydali, Dilara Gundogdu, Artun Bozkurt, Vural Butun, Yesim Soyer, Irem Erel-Goktepe

**Affiliations:** † Department of Biochemistry, 52984 Middle East Technical University, Ankara, Cankaya 06800, Türkiye; ‡ Department of Food Engineering, Middle East Technical University, Ankara, Cankaya 06800, Türkiye; § Department of Chemistry, 52984 Middle East Technical University, Ankara, Cankaya 06800, Türkiye; ∥ Department of Chemistry, Eskisehir Osmangazi University, Eskisehir 26480, Türkiye; ⊥ Center of Excellence in Biomaterials and Tissue Eng, Middle East Technical University, Ankara, Cankaya 06800, Türkiye

## Abstract

There is a need to develop alternative antibacterial agents for antibacterial applications due to the emergence of antibiotic-resistant bacteria. Bacteriophages are natural predators of bacteria and have gained interest as an alternative to antibiotics. The co-delivery of antibacterial agents is an attractive strategy to achieve enhanced antibacterial activity. This study investigated the use of layer-by-layer (LbL) technology to functionalize alginate hydrogels to prepare a *therapeutic* platform, capable of co-delivering *Salmonella* bacteriophage MET P1-001 and curcumin (CUR). First, LbL films consisting of alginate and poly­[2-(diisopropylamino)­ethyl methacrylate]-*block*-poly­[3-dimethyl­(methacryloyloxyethyl)­ammonium propanesulfonate] (PDPA-*b*-βPDMA) micelles were prepared. Multilayers exhibited temperature-responsive behavior through upper critical solution temperature (UCST)-type phase behavior of polyzwitterionic βPDMA coronal chains. LbL assembly temperature affected the film thickness, and the post-assembly temperature was critical to the stability of multilayers against pH changes. Second, these multilayers were deposited on alginate hydrogels containing *Salmonella* bacteriophages, but differently, PDPA-*b*-βPDMA micelles were loaded with CUR. CUR release from hydrogels was greater at pH 5.0 than at pH 7.0. Decreasing the release temperature did not make a considerable effect on the amount of CUR release at pH 7.0 but reduced its release at pH 5.0. The antibacterial activity against *Salmonella enterica subsp. enterica* serovar Enteritidis (*Salmonella* Enteritidis) was mainly due to the release of bacteriophages from the hydrogel and was greater at pH 5.0 than at pH 7.0. Bacteriophages and CUR acted in a combinatorial manner at pH 7.0, while it was not statistically significant at pH 5.0. Overall, this study has generated fundamental knowledge on the preparation of alginate hydrogels co-delivering *Salmonella* bacteriophage MET P1-001 and CUR using LbL technology, and the enhancement of antibacterial efficacy through co-delivery of these therapeutics.

## Graphical abstract

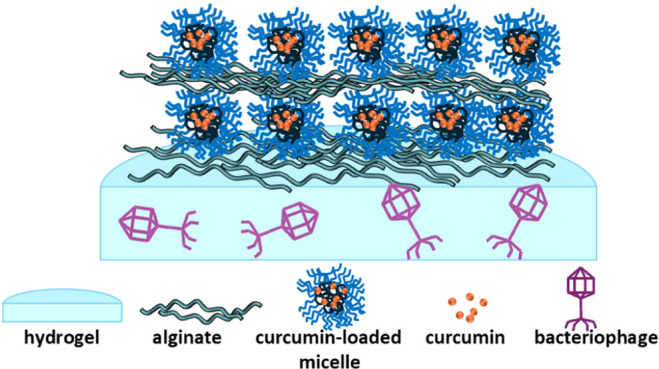

## 1 Introduction

The LbL self-assembly technique involves sequential adsorption of interacting polymers onto a substrate to form multilayer films. Among various thin film deposition methods, such as chemical vapor deposition, Langmuir–Blodgett, selective electrophoresis deposition, and physical vapor deposition, the LbL self-assembly technique has gained prominence because it does not require a complicated equipment;^1^ thus, a smaller investment is sufficient for production infrastructure and energy.^2^ LbL self-assembly can be applied to almost any substrate with varying shape, size, and topography under mild aqueous conditions, making this technique promising, especially for biomedical applications.^3^ In addition, LbL assembly provides control over film composition and thickness at the nanometer scale and allows incorporation/release of a wide range of materials, such as polymers, organic/inorganic nanoparticles, and biologically active molecules, in the multilayers.^4^ One disadvantage of LbL films is that they may exhibit low stability under varying environmental conditions due to their formation commonly through secondary interactions, and therefore crosslinking of multilayers may be needed for certain applications.^5^ LbL films find use in a wide range of areas, such as drug delivery,^6^ tissue engineering,^7^ antibacterial,^8^ and food packaging applications.^9^ The use of block copolymer micelles as building blocks in LbL assembly has been shown to be a promising strategy for increasing the loading capacity of multilayer films for hydrophobic molecules, as the hydrophobic micellar core forms a suitable domain for the deposition of poorly water-soluble drugs.^10^

Polyzwitterions are a class of polymers which contain both positive and negative charges in the same repeating unit and offer high biocompatibility, resistance to protein and cell adsorption, low immunogenicity and stability in biological environments.^11^ The antiadhesive property is provided by the hydration layer formed through hydrogen bonds and ionic solvation between water molecules and the zwitterionic film. Examples to such zwitterionic polymers are polybetaines (polysulfobetaine, polycarboxybetaine, phosphorylcholine), amino-acid containing polymers and copolymers of oppositely charged monomers, so called pseudo zwitterionic polymer.^12^ Self-assembly of block copolymers, composed of polyzwitterionic and hydrophobic blocks, forms micelles with a hydrophobic core and a zwitterionic shell.^13^ Importantly, if the core-forming block is a weak polybase, the formation and dissolution of the micelles can be achieved through pH-trigger, which is of interest especially for anticancer and certain antibacterial applications where drug release at acidic conditions is desired.^14^ For example, dual functional surfaces which effectively prevented bacterial adhesion under physiological conditions while releasing antibacterial agents at moderately acidic pH were prepared using pH-responsive zwitterionic copolymers.^15,16^ Apart from its important biological properties, polyzwitterions such as poly­(sulfobetaine)­s and poly­(phosphorylcholine)­s also exhibit temperature-responsive behavior with an upper critical solution temperature (UCST)-type phase behavior in aqueous medium, thus display coil-to-globule phase transition below the critical point.^17^ Although antiadhesive and/or antibacterial properties of zwitterionic block copolymer micelle based LbL films have been reported, the effect of UCST-type behavior of zwitterionic micelles on the properties of LbL films has not been explored yet. Studies reported so far on LbL films with UCST behavior are based on poly­(acrylamide) (PAAm),^18^ block copolymer micelles of poly­(acrylamide-*co*-acrylonitrile)-*b-*polyvinylpyrrolidone (P­(AAm-*co*-AN)-*b*-PVP),^19^ poly­(quaternized-2-vinylpyridine-*b*-ethylene oxide) (PQ2VP-*b*-PEO),^20^ polyvinylpyrrolidone-*b*-polyureido­(ornithine-*co*-lysine) (PVP-*b*-PUOL),^21^ and a star polypeptide, poly­(l-ornithine-*co*-L-citrulline).^22^ Different from these studies, the first part of this study examined LbL deposition of zwitterionic PDPA-*b*-βPDMA micelles/alginate and the effects of self-assembly and post-assembly temperatures on the formation and the stability of multilayers against pH variations. In the second part of the study, we used these multilayers to modify the surface of alginate hydrogels and design a therapeutic platform that can co-deliver multiple therapeutics for an enhanced antibacterial effect. LbL modification of hydrogels has been reported before for tuning physical and biological properties of hydrogels.^23–28^ On the other hand, the co-delivery of antibacterial agents is an attractive strategy to achieve enhanced antibacterial activity. Herein, we used *Salmonella* bacteriophages as an antibacterial agent and included them in alginate hydrogels. These hydrogels were later used as templates and LbL modified using PDPA-*b*-βPDMA micelles preloaded with curcumin (CUR). Alginate was chosen as the template material due to its biocompatibility, biodegradability, and low cost.^29^ Alginate hydrogels have been widely used for antibacterial applications mainly through incorporation and release of silver nanoparticles or ions,^30^ antibiotics and plant-derived extracts.^31^ Bacteriophages are a promising alternative to traditional antibacterial agents. They are viruses that specifically infect and kill bacteria by injecting their genetic material into bacterial cells. This specificity reduces the risk of disrupting the body’s natural microbiota. Moreover, they can replicate at the site of infection, potentially leading to self-amplifying treatment, which may lower the required dose compared to antibiotics. Bacteriophages are recognized as safe (GRAS) and can be used in the food industry and biomedical therapies without harming the health of human beings. They can also be engineered to overcome bacterial resistance mechanisms, thus offering a dynamic approach for future studies.^32^ Bacteriophages can also disrupt biofilms, so they can be used to treat persistent bacterial infections.^33^ However, bacteriophages can be adversely affected by environmental conditions such as pH, temperature, and UV light exposure, and their viability and activity can be significantly reduced.^34^ Encapsulation of bacteriophages in protective materials has proven to be an effective strategy not only to increase their stability^35^ but also to release them in a controlled manner.^36^ Various polymer platforms have been reported for the integration of bacteriophages to enhance bacteriophage delivery and preserve their infectivity and bioactivity over time.^37–41^ Studies on bacteriophage-containing LbL films have also been carried out, albeit in limited numbers. The infection activities of bacteriophages hosted in multilayer films,^42^ adsorption of bacteriophages on polyelectrolyte multilayers,^43^ and antibacterial efficacy of bacteriophage post-loaded LbL films in food packaging applications have been investigated.^44^ CUR is a natural polyphenolic compound exhibiting anti-inflammatory, antimicrobial and antioxidant properties.^45^ It is poorly soluble in water; therefore, its encapsulation by carrier platforms enhances its bioavailability.^46^ CUR demonstrates efficacy against a wide range of bacterial species, making it a candidate for both biomedical and food applications.^47^ However, its relatively high minimum inhibitory concentration (MIC) can limit its practical use, as such levels may lead to challenges related to formulation, stability, or sensory properties depending on the application matrix.^48,49^ Although synergistic effects have been reported when CUR is combined with antibiotics, the use of antibiotics is prohibited in food systems and subject to strict regulation in animal and human medicine.^50,51^ Therefore, combining CUR with bacteriophages offers a promising alternative approach for the development of effective antibacterial systems in both fields.

In this study, bacteriophage-containing alginate hydrogels, LbL-modified using CUR-loaded PDPA-*b*-βPDMA micelles, were examined with respect to pH/temperature-responsive CUR release and antibacterial activity against *Salmonella* Enteritidis. The combination of CUR with bacteriophages has recently been reported. It was shown that bacteriophage vB_AbaSI_1 combined with CUR effectively killed multidrug-resistant *Acinetobacter baumannii*.^52^ To the best of our knowledge, this is the first study investigating the combined effect of CUR and bacteriophages against *Salmonella*. Overall, this study generated fundamental information on the preparation and characterization of ultrathin multilayer films with pH- and UCST-type temperature-responsive properties and presents the use of LbL technology to modify hydrogels to prepare multifunctional platforms for antibacterial applications.

## 2 Experimental Section

### 2.1 Materials

Sodium alginate (viscosity of 15–25 cP, 1% in H_2_O) (mannuronate/guluronate (M/G) ratio = 1.56) (*M*_*w*_ = 120,000–190,000 Da), ciprofloxacin (≥98%, HPLC), CUR from *Curcuma longa* (>65%), and branched poly­(ethylenimine) (BPEI) (*M*_*w*_ = 25,000 g/mol) were purchased from Sigma-Aldrich. Sulfuric acid (H_2_SO_4_) (98%), sodium hydroxide (NaOH) in the pellet form, and sodium dihydrogen phosphate dihydrate (NaH_2_PO_4_·2H_2_O) were purchased from Merck Chemicals. Calcium chloride anhydrous (CaCl_2_) was obtained from CARLO ERBA Reagents. The purification of deionized (DI) H_2_O was done by passage through a Milli-Q system (Millipore) at 18.2 MΩ. Ethanol (C_2_H_5_OH) (≥99.9%) was purchased from Isolab Chemicals. Luria-Bertani (LB) agar and Brain Heart Infusion (BHI) broth were purchased from Condalab. Mueller–Hinton (MH) agar was purchased from Oxoid.

### 2.2 Synthesis of PDPA-*b*-βPDMA

PDPA_0.33_-*b*-PDMA_0.67_ diblock copolymer was synthesized through group transfer polymerization using sequential monomer addition. The synthetic details of the precursor block copolymer, selective betainization of the DMA block, and their self-assembly behaviors in aqueous solution have been reported before.^53,54^ The molecular weight of the precursor block copolymer was determined by using gel permeation chromatography with an eluent of HPLC-grade THF stabilized with butylated hydroxytoluene (BHT) at a flow rate of 1.0 mL/min. *M*_*n*_ and *M*_*w*_/*M*_*n*_ values were determined as 22,780 g/mol and 1.18, respectively. Calibration was carried out with poly­(methyl methacrylate) standards (Polymer Laboratories). By assuming 100% betainization of the PDMA block, as indicated by the proton NMR spectrum, *M*_*n*_ of the selectively betainized PDPA_0.33_-*b*-βPDMA_0.67_ was calculated as 33,400 g/mol. The GPC chromatogram of PDPA-*b*-PDMA (Figure S1A), NMR spectra of the precursor diblock copolymer PDPA_0.33_-*b*-PDMA_0.67_ (Figure S1B), and the selectively betainized PDPA_0.33_-*b*-βPDMA_0.67_ diblock copolymer (Figure S1C) can be found in the Supporting Information.

### 2.3 Preparation and Characterization of PDPA-*b*-βPDMA Micelles

0.25 mg/mL PDPA-*b*-βPDMA solution was prepared by dissolving PDPA-*b*-βPDMA in 1.0 mM phosphate buffer at pH 3.0. Micellization was induced by gradually increasing the solution pH to 7.0 at 25 °C through dropwise addition of 0.25 M NaOH solution under continuous stirring at 150 rpm. Hydrodynamic size and zeta potential measurements of PDPA-*b*-βPDMA micelles were performed using a Zetasizer Nano-ZS instrument (Malvern Instruments Ltd., U.K.). Hydrodynamic sizes of the particles were obtained by cumulants analysis of the autocorrelation data. The Smoluchowski approximation was used to obtain zeta potential values from electrophoretic mobility. Transmission electron microscopy (TEM) imaging of PDPA-*b*-βPDMA micelles was performed at an acceleration voltage of 20–120 kV using FEI Tecnai G2 Spirit Bio-Twin CTEM.

### 2.4 Preparation of Multilayers of Alginate and PDPA-*b*-βPDMA Micelles

Silicon wafers were treated with acetone for 10 min at 50 °C. Then, the wafers were immersed into methanol at room temperature for 3–4 min, rinsed with DI water, and dried under N_2_ flow. Next, the wafers were treated with concentrated H_2_SO_4_ for 85 min, rinsed with DI water and dried under N_2_ flow. Lastly, the wafers were treated with 0.25 M NaOH solution for 10 min, washed with DI water, and dried under N_2_ flow.

BPEI was dissolved in 10.0 mM phosphate buffer (at pH 5.5) at a 0.5 mg/mL concentration overnight. Alginate was dissolved in 1.0 mM phosphate buffer (at pH 7.0) at a 0.25 mg/mL concentration overnight. PDPA-*b*-βPDMA was dissolved overnight in 1.0 mM phosphate buffer at pH 3.0 and self-assembled into micelles prior to LbL construction, as described in Section 2.3. A single layer of BPEI was deposited onto clean silicon wafers as a precursor layer prior to multilayer deposition. For this purpose, the clean wafers were immersed into BPEI solution (prepared in 10.0 mM phosphate buffer at pH 5.5) for 30 min at pH 5.5, followed by rinsing twice with 10.0 mM phosphate buffer at pH 5.5 for 2 min and drying under N_2_ flow. For multilayer deposition, BPEI-coated wafers were first immersed into 0.25 mg/mL alginate solution for 15 min at pH 7.0, rinsed twice with 1.0 mM phosphate buffer (pH 7.0) for 2 min, and dried under N_2_ flow. The substrate was then immersed into 0.25 mg/mL PDPA-*b*-βPDMA micelle solution for 15 min at pH 7.0, rinsed twice with 1.0 mM phosphate buffer at pH 7.0 for 2 min, and dried under N_2_ flow. Deposition of alginate and PDPA-*b*-βPDMA micelles was continued until the desired number of layers had been constructed at the substrate. Dry thickness values were measured using a spectroscopic ellipsometer (Optosense, OPT-S6000). For LbL film preparation at 17 °C and 22 °C, the temperature of the assembly solutions was adjusted prior to deposition and maintained constant throughout the process. The average film thickness was determined by measuring three different regions on the surface of each silicon wafer. The error bars correspond to the standard deviation (SD) calculated from three measurements per sample.

### 2.5 Stability of Alginate/PDPA-*b*-βPDMA Micelle Multilayers

12-layers of PDPA-*b*-βPDMA micelles/alginate films (prepared at either 25 °C or 17 °C) were immersed into 10.0 mM phosphate buffer at decreasing and increasing pH values at 7 °C, 25 °C, and 37 °C. The immersion time at each pH was 30 min. The stability of the films was evaluated by monitoring the fraction retained at the surface against pH. The fraction retained on the surface was calculated as the thickness of the films after exposure to a buffer solution at a given pH divided by the initial film thickness. The average film thickness was determined by measuring three different regions on the surface of each silicon wafer. The error bars correspond to the SD values calculated from three measurements per sample.

### 2.6 Preparation of Bacteria and Bacteriophage Suspensions

As a model pathogen, a *Salmonella enterica subsp. enterica* serovar Enteritidis (*Salmonella* Enteritidis) strain, MET S1-001, was used. MET S1-001 (*Salmonella* Enteritidis) was previously isolated from chicken meat and stored at −80 °C.^55^ MET S1-001 is susceptible to amikacin, amoxicillin-clavulanic acid, ampicillin, chloramphenicol, ciprofloxacin, gentamicin, ceftriaxone, ceftiofur, ertapenem, cefoxitin, imipenem, kanamycin, cephalothin, nalidixic acid, streptomycin, sulfisoxazole, sulfamethoxazole-trimethoprim, and tetracycline.^55^ MET S1-001 was used as the target host for bacteriophage infection and was cultured on BHI medium before the experiments.

*Salmonella* Enteritidis bacteriophage MET_P1_001_43, which is a lytic bacteriophage with a 43,205 bp genome, was used as an antimicrobial agent. MET_P1_001_43 was previously isolated from cattle manure and stored at 4 °C.^56^ The genome sequence of MET_P1_001_43 is available on NCBI (accession number: OP389270). Before the experiments, bacteriophage solutions were freshly prepared and adjusted to 10^8^ plaque-forming units per milliliter (PFU/mL) using 10.0 mM phosphate buffer at pH 7.0 and confirmed by double plaque assay (DPA). For the bacteriophage MET-P1-001, MOI and lysis assays against *Salmonella* were conducted in one of our previous studies, and its antibacterial effect was evaluated. As shown in the supplementary file of the referred publication, a reduction in bacterial cell counts was observed even when the applied bacteriophage concentration was as low as 1 log PFU/mL.^56^

### 2.7 Enumeration of Bacteriophage

To determine the bacteriophage counts, DPA was conducted in each experiment. The bacteriophage solution was serially diluted by using 10.0 mM phosphate buffer at pH 7.0. LB (1.5%) agar was used as the base agar. To LB top agar (0.6%), 100 μL of the host bacterium (MET S1-001) and 100 μL of the corresponding dilution of the bacteriophage solution (MET_P1_001_43) were added, gently swirled, and poured onto the LB base agar. After waiting for the top agar to solidify for 10 min on the bench, the plates were incubated at 37 °C for about 20 h. Afterwards, the plaques on the Petri dish were counted and multiplied with dilution factors, and the bacteriophage concentration in the original 10.0 mM phosphate buffer solution at pH 7.0 was determined.

### 2.8 Preparation of Alginate Hydrogels

20 mg of alginate was added into 0.5 mL of either of the following solutions: (i) bacteriophage solution (prepared in 10.0 mM phosphate buffer at pH 7.0), (ii) 10.0 mM phosphate buffer at pH 7.0 (for negative control), and (iii) 0.025 mg/mL ciprofloxacin solution at pH 7.0 (for positive control). Of note, ciprofloxacin was dissolved in 10.0 mM phosphate buffer at pH 2.5, and then the pH was raised to 7 prior to use. The solutions were stirred at ∼1000 rpm for 1 h. To create disk-shaped hydrogels, the viscous pre-gel solution was poured into a mold and submerged into 40.0 mL of 0.4 M CaCl_2_ solution (dissolved in DI water) for 20 min. Afterwards, the hydrogels were submerged into 40.0 mL of DI water for 20 min to remove excess CaCl_2_. The prepared hydrogels were wrapped in Al foil and kept overnight at 25 °C.

### 2.9 CUR Loading into PDPA-*b*-βPDMA Micelles and Preparation of LBL Coated Bacteriophage Containing Alginate Hydrogels

PDPA-*b*-βPDMA was dissolved overnight in a 1 mM phosphate buffer solution at pH 3.0 at a concentration of 1.05 mg/mL. 2.0 mg/mL CUR solution was prepared in ethanol and diluted to ∼0.3 mg/mL by adding pure ethanol. The CUR solution was added into PDPA-*b*-βPDMA solution so that the final concentrations of PDPA-*b*-βPDMA and CUR were 1.0 and 0.015 mg/mL, respectively. Micellization was triggered with the increase of pH from 3.0 to 7.0 by dropwise addition of 0.25 M NaOH solution under stirring at 150 rpm and 25 °C. The micelle solution was kept overnight under constant stirring for CUR loading into the micelles. Hydrodynamic size measurements were conducted using Zetasizer Nano-ZS equipment (Malvern Instruments Ltd., U.K.). The emission spectra of CUR in the absence and presence of PDPA-*b*-βPDMA unimers/micelles were recorded by using a PerkinElmer LS55 Fluorescence Spectrometer. CUR was excited at 425 nm with the 5 and 2.5 nm excitation and emission slit widths, respectively.

Bacteriophage-containing alginate hydrogels were LbL-modified at pH 7.0 and 25 °C using the procedure described in Section 2.4 except for the concentrations of micelle and alginate solutions. 1.0 mg/mL micelle and alginate solutions were used in the hydrogel modification. Scanning Electron Microscopy (SEM) images of the hydrogels were obtained using a JSM-6400 Scanning Electron Microscope.

### 2.10 CUR Release from PDPA-*b*-βPDMA Micelle/Alginate Multilayers and LBL Modified Alginate Hydrogels

CUR release was followed from multilayers (40-layer) on glass slides and 10-layer modified alginate hydrogels separately by immersing them into 10.0 mM phosphate buffer under the following conditions: pH 7.0/7 °C; pH 7.0/25 °C; pH 5.0/7 °C; pH 5.0/25 °C. 1.0 mL from the release solution was taken after every 1 h and diluted at a 1:1 ratio by volume using pure ethanol. The fluorescence intensity of CUR was followed using a PerkinElmer LS55 Fluorescence Spectrometer (excitation/emission wavelength: 425/535 nm; excitation/emission slit widths: 10/10 nm). Of note, 1.0 mL of 10.0 mM phosphate buffer at the same pH and temperature was added to the release solution to keep the volume constant through the whole process. CUR was quantified using calibration curves that were prepared under release conditions. The average CUR concentration was determined by using three independent hydrogel replicates. The error bars correspond to the SD calculated from the concentration of released CUR from each hydrogel.

### 2.11 Bacteriophage Release from LbL Coated Hydrogels

The procedure that was reported earlier by Khazani Asforooshani et al.^57^ was followed with minor modifications. 50 mL of LB broth was prepared in an Erlenmeyer flask. For the experiment conducted at pH 5.0, the pH was adjusted. Then, 100 μL of bacterial culture (OD_600_ = 0.1) was inoculated to the flask. Bacteriophage-loaded and LbL-modified hydrogels were incubated in the culture overnight at 37°C while being shaken at 150 rpm. The control group contained only bacteria. After incubation, the OD_600_ value was measured to monitor bacterial growth. Error bars represent the SD of the mean, calculated based on measurements from three independent hydrogel replicates.

### 2.12 Kirby–Bauer/Disk-Diffusion Test

The inoculum was prepared in MH broth in the log phase. The concentration of the inoculum was adjusted with sterile 10.0 mM phosphate buffer (pH 7.0) to 0.5 McFarland standard using a densitometer (DEN-1B). It was used within 15 min after preparation. Bacteria were inoculated onto MH agar with a sterile swab by streaking it three times. The manufacturer’s instructions were followed to prepare the MH agar plates. The pH was adjusted to either 7.0 or 5.0. Among five different hydrogel samples, one of them was a blank (bare alginate hydrogel) and used as a negative control, and one of them was ciprofloxacin-encapsulated alginate hydrogel and used as a positive control. The remaining three samples (10-layer coated bacteriophage-containing hydrogel) were used as replicates for the disk-diffusion test. The samples were placed in the center of the agar plate. Afterwards, each agar plate was incubated at 37 °C for about 18 h. The GelDoc Go Imaging System (Bio-Rad, USA) was used for the visualization of the zones. Zone images were taken using the GelDoc Go Imaging System (Bio-Rad, USA). Error bars represent the SD of the mean, calculated based on measurements from three independent hydrogel replicates.

### 2.13 Statistical Analysis

Minitab was used to perform statistical analyses. The means of zone sizes were compared using a one-way ANOVA test. Differences were taken into account as statistically significant when *p* < 0.05.

## 3 Results and Discussion

PDPA-*b*-βPDMA is a zwitterionic block copolymer composed of zwitterionic βPDMA and pH-responsive PDPA blocks (Scheme 1A). pH-triggered micellization of PDPA-*b*-βPDMA has been reported in our previous studies.^15,16^ Briefly, spherical micelles with a diameter of ∼25 nm were prepared by dissolving PDPA-*b*-βPDMA at an acidic pH and room temperature, followed by gradually increasing the solution pH to 7.0. The driving force for the formation of self-assembled micelles with PDPA core and zwitterionic βPDMA corona was deprotonation of amino groups of PDPA blocks with increasing pH and enhanced hydrophobic association among PDPA chains. The zeta-potential of micelles at pH 7.0 was recorded as +7.15 mV. Considering the electrically neutral zwitterionic corona, the positive zeta potential was attributed to the partially protonated tertiary amino groups that remained at the surface of PDPA micellar core blocks. Of note, micelles precipitated above pH 7.0. Therefore, the micellization pH was optimized to 7.0. The hydrodynamic size distribution, TEM image, and zeta potential distribution of PDPA-*b*-βPDMA micelles are presented in Figure S2.

**Scheme 1:**
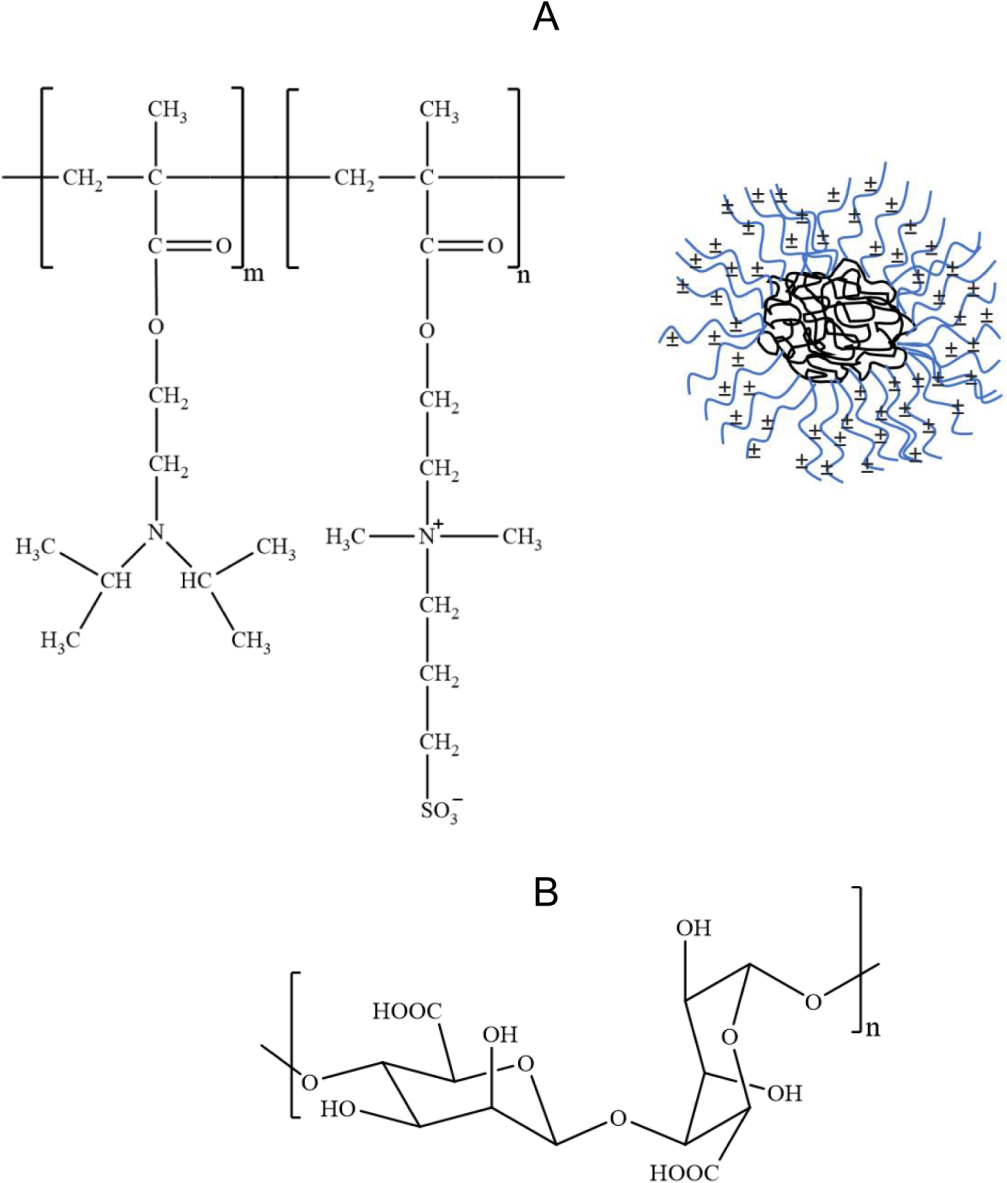
(A) Chemical Structure of PDPA-*b*-βPDMA and Schematic Representation of PDPA-*b*-βPDMA Micelles with Zwitterionic βPDMA-Corona and PDPA-Core. (B) Chemical Structure of Alginate

### 3.1 LbL Deposition and Stability of Multilayers

#### 3.1.1 LbL Deposition of Alginate and PDPA-*b*-βPDMA Micelles

Alginate (Scheme 1B) was used as the negatively charged polymer for the LbL deposition of PDPA-*b*-βPDMA micelles at pH 7.0. p*K*_a_ values of mannuronic acid and guluronic acid units of alginate are 3.38 and 3.65, respectively.^58^ Multilayer deposition was performed at pH 7.0 and 25 °C. The driving force for LbL growth was mainly electrostatic interactions between carboxylate groups of alginate and quaternized amino groups of βPDMA coronal chains, together with tertiary amino groups at the surface of the PDPA core. Besides, hydrogen bonding interactions between sulfonate groups of βPDMA corona and hydroxyl groups of alginate might have also contributed to the film growth. The evolution of film thickness as a function of layer number is shown in Figure 1A. A zigzag growth profile was observed, with a decrease in thickness due to desorption from the surface during alginate deposition and an increase in thickness after PDPA-*b*-βPDMA micellar layer deposition. The solubilization of PDPA-*b*-βPDMA micelles in an excess amount of alginate was attributed to the high solubility of alginate in water. Apart from this, polyelectrolyte multilayers display a so called “stripping” behavior when one of the polymer pairs has a relatively low molecular weight.^59^ The molecular weight of the βPDMA corona block was considerably lower than that of alginate. While alginate deposited at the surface, PDPA-*b*-βPDMA micelles might have partially desorbed from the surface, forming water-soluble complexes with alginate. As a result, a decrease in thickness was observed upon the deposition of each alginate layer.

**Figure 1:**
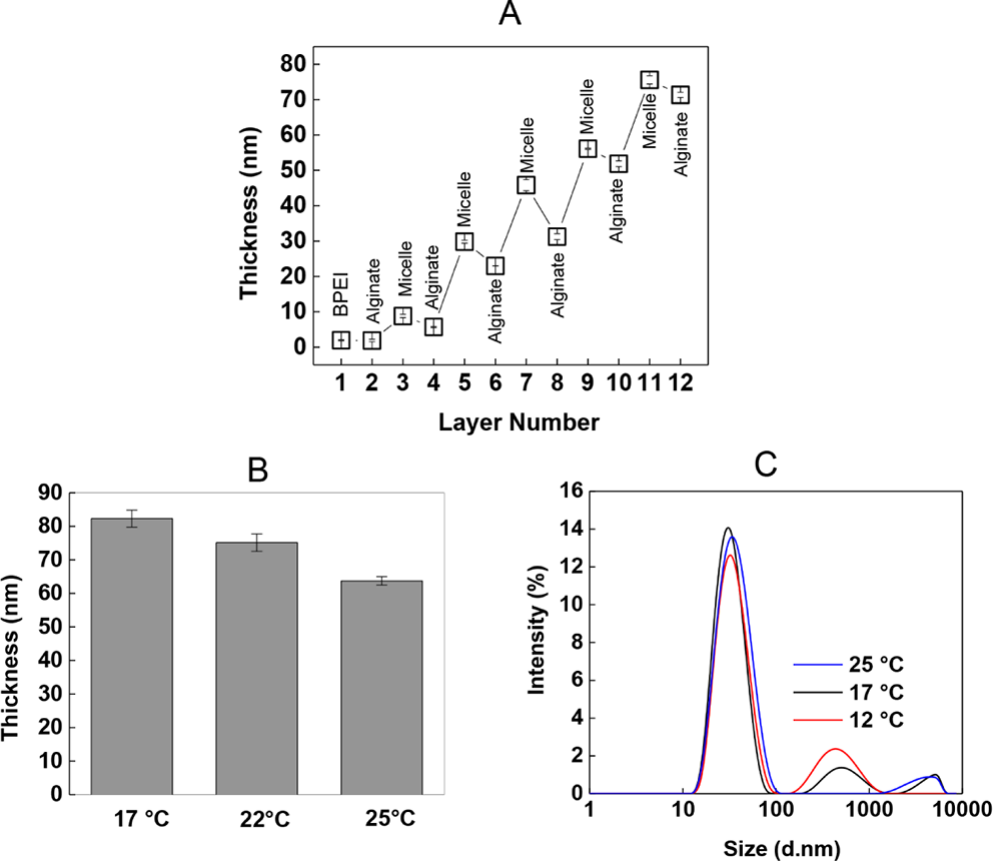
(A) LbL growth of PDPA-*b*-βPDMA micelles and alginate at pH 7.0 and 25 °C. (B) Comparison of ellipsometric thickness of 12-layers of PDPA-*b*-βPDMA micelles/alginate films, prepared at 17 °C, 22 °C, and 25 °C. Concentrations of PDPA-*b*-βPDMA micelles and alginate were both 0.25 mg/mL. (C) The size distributions (by intensity) of PDPA-*b*-βPDMA micelles (1.0 mg/mL, prepared at pH 7.0 and 25 °C) after exposure to 17 and 12 °C separately for 1 h. The distribution at 25 °C was plotted for comparison.

The effect of LbL deposition temperature on the multilayer growth was examined by comparing the ellipsometric thickness of 12-layer PDPA-*b*-βPDMA micelles/alginate films prepared at 17 °C, 22 °C, and 25 °C. The highest film thickness was obtained at 17 °C, while the lowest thickness was recorded at 25 °C (Figure 1B). The increasing film thickness with decreasing temperature can be explained by the deposition of βPDMA chains at the surface in a more compact conformation due to the transition of βPDMA chains from extended to globular form at temperatures close to its UCST. Besides, enhanced hydrophobicity of βPDMA coronal chains with decreasing temperature possibly promoted hydrophobic–hydrophobic association between the micelles and alginate, leading to a greater amount of deposition at the surface. Of note, the zwitterionic βPDMA block has a UCST around 15 °C.^60^ The increasing hydrophobicity of PDPA-*b*-βPDMA micelles with decreasing temperature was indirectly confirmed through intensity average size measurements using the DLS technique. Figure 1C compares the size distribution at 25 °C, 17 °C, and 12 °C. The emergence of an additional peak at higher size values became slightly more remarkable at 12 °C, indicating the presence of larger aggregates in the solution. The formation of larger aggregates was correlated with the enhanced association among zwitterionic coronal chains near the critical temperature as the zwitterionic βPDMA block became more hydrophobic. The increase in multilayer thickness as the critical temperature approached has been reported in the literature for LbL films obtained from polymers with a lower critical solution temperature (LCST)-type phase behavior. For example, LbL deposition of poly­(methacrylic acid) (PMAA) and poly­(*N*-isopropylacrylamide) (PNIPAM) with an LCST of ∼32 °C yielded ∼4.4 nm, ∼6.7 nm, and ∼8.7 nm at 10 °C, 23 °C, and 30 °C, respectively. This result was correlated with the worsening of the solvent quality and more compact conformation of PNIPAM chains as the deposition temperature approached the LCST of PNIPAM.^18^

#### 3.1.2 Stability of Alginate/PDPA-*b*-βPDMA Micelle Multilayers

##### 3.1.2.1 Effect of Temperature on the Stability of the Multilayers

The effect of the temperature on film stability was investigated from two different perspectives:

i) Effect of post-assembly temperature on the stability of multilayers which were constructed at 25 °C.
ii) Effect of LbL self-assembly temperature on the stability of multilayers.

For the first case (i), LbL films were prepared at pH 7.0 and 25 °C and exposed separately to decreasing and increasing (Figure 2A) pH at three different temperatures, i.e., 7 °C, 25 °C, and 37 °C. The stability at 7 °C was examined because, in general, food products are stored and transported under refrigerated conditions, generally within the temperature range of 0–10 °C.^61^ On the other hand, stability at 37 °C was examined not only because it is an optimal temperature for growing bacteria in the antibacterial tests^62^ but also because it is in the application temperature range for the biomedical field.^63^ The stability at 25 °C was conducted for comparison. The stability of the films was evaluated by monitoring the fraction retained at the surface against pH. The fraction retained on the surface was calculated as the thickness of the films after exposure to buffer solution at a given pH divided by the initial film thickness. Although the multilayers were deposited at pH 7.0/25 °C, thickness loss was detected when the films were exposed to pH 7.0/25 °C (∼6% thickness loss) and pH 7.0/37 °C (∼15% thickness loss) for 30 min. This is the reason for the fraction being lower than unity at pH 7.0 at 25 and 37 °C. At decreasing pH (Figure 2A), the lowest film stability was observed at 37 °C. Multilayers were erased completely from the surface at pH 5.0. The factors affecting the dissolution of the films can be listed as follows: (i) the disruption of electrostatic interactions among the layers with increasing temperature and (ii) the excess amount of charge created within the multilayers due to protonation of tertiary amino groups of PDPA, followed by the disintegration of the micellar cores as the pH was decreased below p*K*_a_ (∼6.4) of PDPA. Of note, on the contrary, the carboxylate groups of alginate protonate with decreasing pH. Therefore, excess positive charge might not be effectively compensated by alginate, and the electrostatic repulsion between PDPA chains caused destabilization of the multilayers. In addition, the osmotic pressure of the counterions which penetrated the multilayers to compensate for the excess charge on PDPA chains was possibly followed by diffusion of water molecules and swelling of the film, which eventually led to complete dissolution of the layers. Stability at 25 °C was relatively higher than that at 37 °C, confirming the adverse effect of increasing temperature on the electrostatic and hydrogen bonding-driven association between the layers. The thickness decreased gradually at pH below the p*K*_a_ of PDPA and ∼30% of thickness loss was recorded under strongly acidic conditions. The highest film stability was obtained at 7 °C. The film thickness almost remained the same within the whole pH range. In addition to electrostatic association among the layers, which strengthened with decreasing temperature, the increased hydrophobicity of βPDMA chains as the temperature dropped below the critical point might have brought additional stability to the multilayers. The effect of hydrophobicity of the polymers on multilayer stability has been reported before.^64^

**Figure 2:**
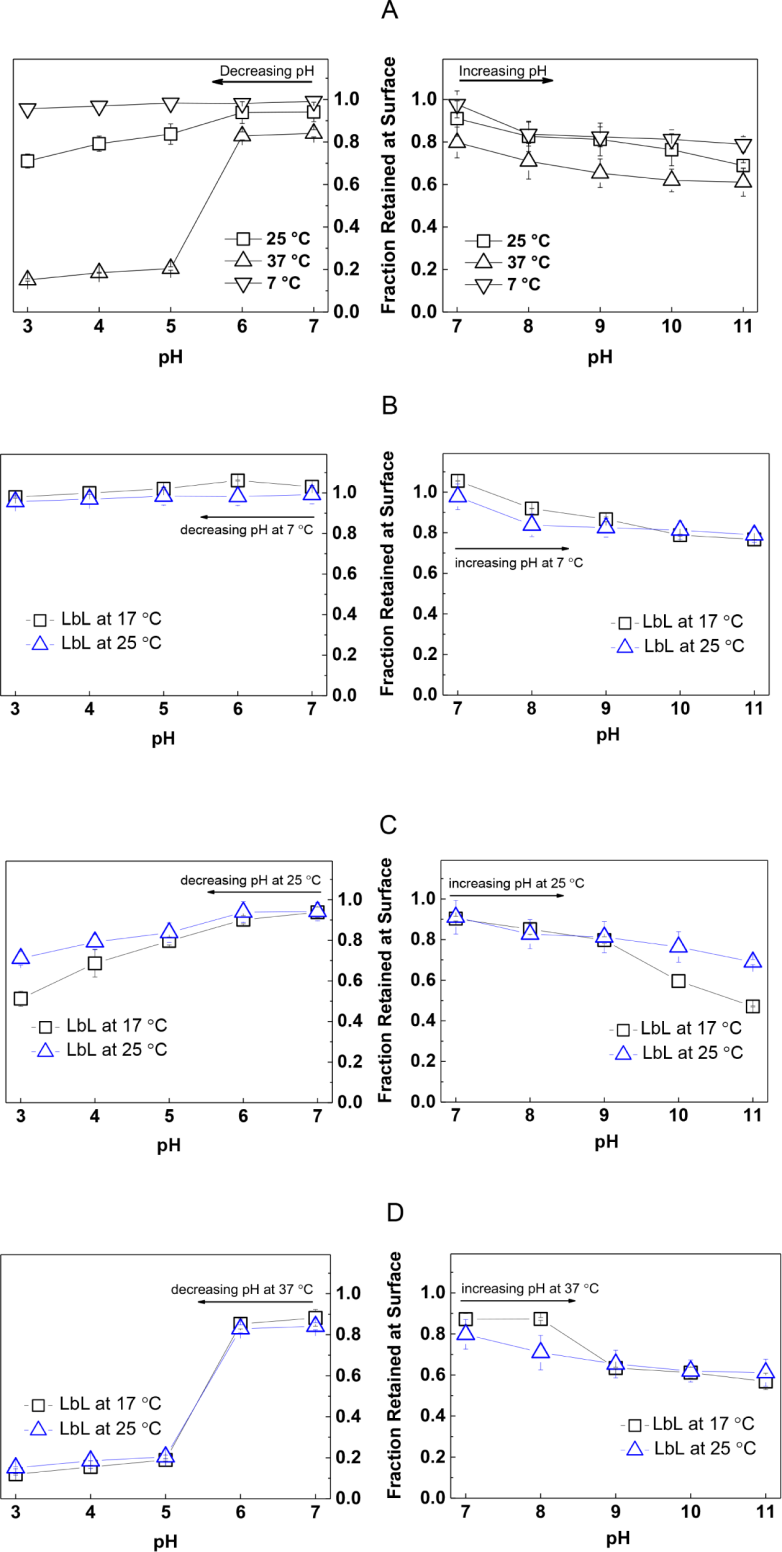
(A) Fraction retained at the surface of 12-layer PDPA-*b*-βPDMA micelles/alginate film (prepared at pH 7.0/25 °C) after exposure to 10.0 mM phosphate buffer solution at 7 °C, 25 °C, and 37 °C under decreasing and increasing pH conditions. Comparison of the pH stability of the films prepared at pH 7.0/17 °C and pH 7.0/25 °C. Fraction retained at the surface of 12-layer PDPA-*b*-βPDMA micelles/alginate film (prepared separately at pH 7.0/17 °C and pH 7.0/25 °C) after exposure to 10.0 mM phosphate buffer solution at (B) 7 °C, (C) 25 °C, and (D) 37 °C under decreasing and increasing pH conditions. Exposure time at each pH was 30 min.

At increasing pH (Figure 2A), similar to the behavior of the films at decreasing pH, the fraction of the film retained at the surface decreased with increasing temperature. However, the thickness loss from the surface was lower at basic conditions compared to that recorded at acidic pH. This result suggests that the excess positive charge generated within the multilayers by decreasing pH was more critical for the dissolution of the films than the decrease in interlayer association due to increasing temperature. The effect of post-assembly temperature on the stability of hydrogen bonding-driven PAAm/PMAA LbL films with UCST behavior has been examined. The disintegration pH of multilayers was found to decrease as the post-assembly temperature increased from 10 to 37 °C, since at higher temperatures the solubility of PAAm enhanced and the hydrogen bonding among PAAm/PMAA layers weakened.^18^ In another study, polyvinylpyrrolidone-*b*-polyureido­(ornithine-*co*-lysine) (PVP-*b*-PUOL) self-assembled into micelles with polypeptide PUOL core and PVP corona below the UCST (33 °C) of the PUOL block. LbL films of PVP-*b*-PUOL micelles and TA exhibited temperature-induced swelling/deswelling behavior when exposed to temperatures above (40 °C) and below (25 °C) the UCST of the PUOL block due to the increase/decrease of the micelle diameter.^21^

Increasing film stability with decreasing temperature raised the question of whether the LbL self-assembly temperature would have an effect on the film stability. For this purpose, LbL films were prepared at pH 7.0/17 °C and exposed to decreasing or increasing pH at 7 °C (Figure 2B), 25 °C (Figure 2C), and 37 °C (Figure 2D). Although LbL assembly at 17 °C provided thicker films, it did not contribute to the stability of the films at decreasing pH values at all post-assembly temperatures. On the other hand, deposition at 17 °C provided slightly higher stability at basic conditions only up to pH 9.0.

### 3.2 LbL Self-Assembly of CUR Loaded PDPA-*b*-βPDMA Micelles onto Bacteriophage Containing Hydrogels

#### 3.2.1 Preparation of Bacteriophage Containing Alginate Hydrogels

Characterization of bacteriophages through TEM imaging, hydrodynamic size, and zeta potential measurements has been reported in our previous study.^44^ In this study, TEM imaging and hydrodynamic size analyses were performed again before the experiments (Figure S3). Briefly, TEM imaging clearly showed the head and long tail parts of the bacteriophages. The zeta potential of the bacteriophages at pH 7.0 was measured as ∼−8.5 mV, indicating that carboxyl and any other negatively charged functional groups were dominating the outer shell of the bacteriophages at pH 7.0. The hydrodynamic size distribution by intensity was uniform with a single peak, and the average size of the bacteriophages was ∼180 nm. Alginate hydrogels used in this study have been previously synthesized and characterized in our laboratory.^25^ For the encapsulation of bacteriophages, alginate was dissolved in bacteriophage solution (pH 7.0) at a concentration of 40 mg/mL and poured into a mold. Gelation was triggered by immersing the mold into a 0.4 M CaCl_2_ solution. Hydrogels were formed through electrostatic interactions between the chelating Ca^2+^ ions and COO^–^ groups.^65^ Hydrogel formation was reported to occur through guluronic acid units of alginate, which created a cavity-like egg-box shape and accommodated divalent cations (e.g., Ca^2+^).^66^ Figure 3A compares top-view SEM images of bare and bacteriophage-containing alginate hydrogels. The surface of bacteriophage-loaded hydrogels exhibited increased roughness compared to that of bare hydrogels. The size of the spherical aggregates on the hydrogel surface was in good agreement with the size of the bacteriophage head, indicating that bacteriophages were located both inside and on the hydrogel surface. A zoomed-in image is presented in the inset to recognize the bacteriophages. Cross-sectional SEM images (Figure 3B) showed that the bacteriophage-loaded hydrogels had a denser internal structure. When the cross-sectional SEM images were zoomed in, bacteriophages within the hydrogel could also be seen. Of note, we first started with alginate hydrogels prepared at an alginate concentration of 80 mg/mL. However, no antibacterial activity was observed with these gels, probably because the dense hydrogel structure prevented the diffusion of bacteriophages out of the hydrogel template. Therefore, we continued our studies with bacteriophage-containing alginate hydrogels prepared at a concentration of 40 mg/mL. However, it should be noted that as the number of layers deposited on the hydrogel surface increases during the LbL process, the hydrogels become mechanically weakened due to the partial loss of alginate chains from the hydrogel. This makes handling the hydrogels difficult. This problem becomes more prominent, especially as the alginate concentration in the gel decreases. Therefore, we optimized the number of layers in the preparation of LbL-modified hydrogels to 10.

**Figure 3:**
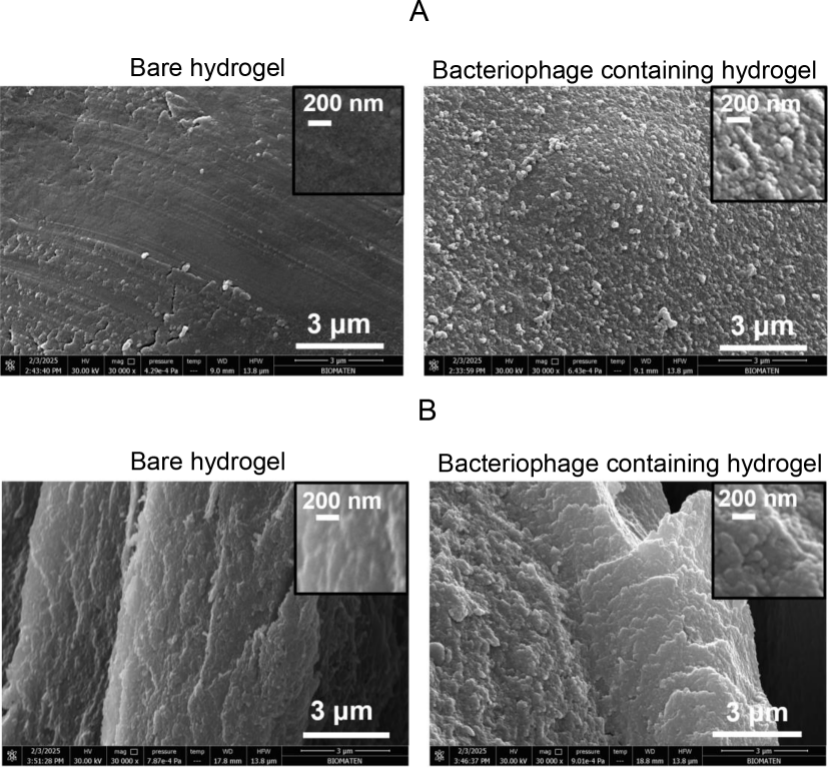
(A) Top view and (B) cross-sectional SEM images of bare and bacteriophage-containing alginate hydrogels. Insets show zoomed-in images.

#### 3.2.2 CUR Loading into PDPA-*b*-βPDMA Micelles

CUR is a natural compound obtained from turmeric rhizomes rhizomes (*Curcuma longa* L.). It is a biologically important molecule because it is nontoxic, biodegradable, and shows anti-inflammatory, anti-hypertensive, antioxidant, and anti-cancer properties.^67^ In recent years, the use of CUR as an antioxidant and antimicrobial agent in the design of smart food packaging has attracted attention. Importantly, CUR can be used in the preparation of edible packaging since it is both nontoxic and biodegradable.^68^ In addition to the important biological properties of CUR, the fact that its mechanism of action has been elucidated makes it preferred in food and biomedical applications. For example, CUR responds to the pH changes in food. While it is yellow between pH 3.0–7.0, it shifts from yellow to red as the pH increases from 7.0 to 8.0. Therefore, CUR is of interest for food packaging applications not only because of its biological properties but also because consumers can have an idea about the freshness of the food by observing the food color changes, even with the naked eye.^69^ CUR is frequently used in the food industry and is approved by the World Health Organization (WHO) and Food and Drug Administration (FDA).^70^ Use of CUR is challenging in certain applications due to its low water solubility and rapid degradation. CUR stability can be increased with the use of micro-/nano-encapsulation systems.^71^ In this respect, block copolymer micelles are suitable nanostructures to encapsulate CUR in the hydrophobic micellar cores and increase its solubility.

CUR solution (0.3 mg/mL in ethanol) was added into PDPA-*b*-βPDMA aqueous solution so that the final concentrations of PDPA-*b*-βPDMA and CUR were 1.0 and 0.015 mg/mL, respectively. Unfortunately, we could not further increase the concentration of CUR solution used for loading into micelles. The solubility of CUR in water is low, and we could increase its concentration with increasing the amount of ethanol in the buffer. However, increasing the ethanol amount adversely affected the solubility of the polymer in the solution. To decide the maximum amount of ethanol in the polymer solution, we followed the hydrodynamic size of PDPA-*b*-βPDMA as a function of the increasing ethanol concentration in the buffer before micellization. A significant increase in hydrodynamic size of PDPA-*b*-βPDMA was recorded when the volume percent of ethanol exceeded 5% in the buffer, indicating aggregation of PDPA-*b*-βPDMA. Therefore, we optimized the volume percent of ethanol as 5%, and the CUR concentration was determined accordingly. Micellization was triggered by increasing the solution pH to 7.0. The micellar solution was stirred at room temperature overnight to allow CUR loading into micellar cores. Given the hydrophobic–hydrophobic interactions between CUR and micellar cores, it is suggested that CUR was mainly loaded into the cores of micelles. However, some amount of CUR might have also adsorbed on the βPDMA coronal chains through hydrophobic interactions between the polymer backbone and CUR. The size of CUR-loaded micelles (average size ∼32 nm) was slightly larger than that of bare micelles (average size ∼25 nm) (Figure 4A). The increase in size may be due to the increased aggregation of PDPA-*b*-βPDMA unimers, owing to the enhanced hydrophobic interactions provided by CUR adsorbed on the polymer chains.

**Figure 4:**
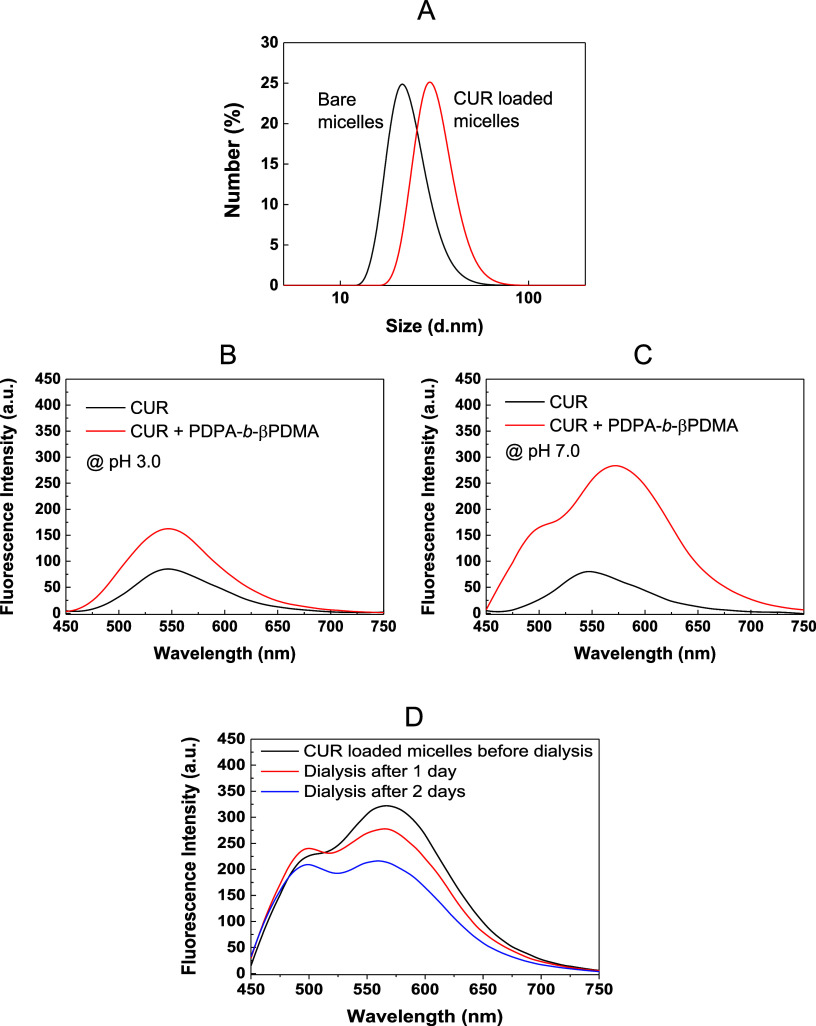
(A) Comparison of hydrodynamic size distributions of bare and CUR loaded micelles at pH 7.0. Emission spectra of CUR in the presence and absence of PDPA-*b*-βPDMA at (B) pH 3.0 and (C) pH 7.0. (D) Emission spectra of CUR in samples taken from the micelle solution (pH 7.0) before and after dialysis.

Next, CUR loading into micelles was examined by using fluorescence spectroscopy. Figure 4B,C compares the emission spectrum of CUR in the presence and absence of PDPA-*b*-βPDMA at pH 3.0 and pH 7.0, respectively. CUR has a broad peak at 560 nm at pH 3.0. The intensity of the peak increased in the presence of PDPA-*b*-βPDMA, suggesting association of CUR and polymer chains, enhancing its solubility (Figure 4B). On the other hand, the difference in the intensity of the peaks at 560 nm became more remarkable at pH 7.0 when PDPA-*b*-βPDMA transformed into micellar aggregates (Figure 4C). Besides, a shoulder emerged around 495 nm at pH 7.0. Raveendran et al. encapsulated CUR in block copolymer micelles with a PCL core and Pluronic corona and reported that CUR had a weak broad peak at 550 nm, while a well-defined high-intensity blue-shifted fluorescent peak was recorded at 495 nm when CUR was encapsulated by micelles. They explained this shift in the peak position by the encapsulation of CUR within the micellar cores.^72^ In another study, in addition to the peak at 548 nm, a shoulder at 488 nm was realized in the emission spectrum of CUR at pH 2.0. In this study, there was no polymer in the solution, and this shoulder was correlated with the formation of CUR aggregates.^73^ In light of these studies, the peak at 495 nm was correlated with the encapsulated CUR in the micellar core, while the peak at 560 nm was associated with the free CUR. In other words, the solution contained free CUR, which remained unencapsulated and co-existed with CUR-containing PDPA-*b*-βPDMA micelles. To confirm this, the micellar solution was dialyzed against buffer (pH 7.0) to remove free CUR. Figure 4D compares the emission spectra of CUR before and after dialysis. As the free CUR was removed from the micellar solution, the ratio of the intensity at 495 nm to 560 nm increased, supporting the existence of free CUR in the solution. However, the peak at 560 nm did not disappear even after 2 days of dialysis. Therefore, LbL deposition onto bacteriophage-containing alginate hydrogels was proceeded without dialysis of the micellar solutions.

#### 3.2.3 Multilayer Deposition on Bacteriophage Containing Alginate Hydrogels

Bacteriophage-containing alginate hydrogels were LbL modified using CUR-containing PDPA-*b*-βPDMA micelles and alginate. Of note, alginate was used for two different purposes: the first one is the production of a hydrogel template, and the second one is the deposition of micelles on the surface by the LbL technique. Although LbL deposition at pH 7.0 and 17 °C provided the thickest films, LbL deposition onto bacteriophage-containing alginate hydrogels was performed at pH 7.0 and 25 °C. Our previous studies have shown that LbL building blocks also diffuse into the hydrogel matrix during multilayer deposition at the surface.^24,25^ For this reason, we chose to establish relatively weak interactions between the alginate chains to include CUR-containing PDPA-*b*-βPDMA micelles not only on the surface but also inside the hydrogel to further contribute to antibacterial activity. Figure 5A presents photographs of 10-layer and 30-layer coated hydrogels. The darkening of the hydrogel color was due to the increase in the amount of CUR-loaded micelles deposited in the hydrogel and on its surface as the number of layers increased. Of note, bacteriophage-containing alginate hydrogels are colorless, and the yellow color of the hydrogels is due to the CUR. For SEM imaging (Figure 5B), 30 layers were deposited on the hydrogel so that the modification could be clearly observed. The 30-layer modified bacteriophage-containing hydrogel had a more porous structure, as seen in the top-view and cross-sectional images. This can be attributed to the partial release of alginate and bacteriophages from the hydrogel during the LbL modification. Considering that bacteriophage loss may adversely affect the antibacterial activity, the number of layers was limited to 10 for further studies.

**Figure 5:**
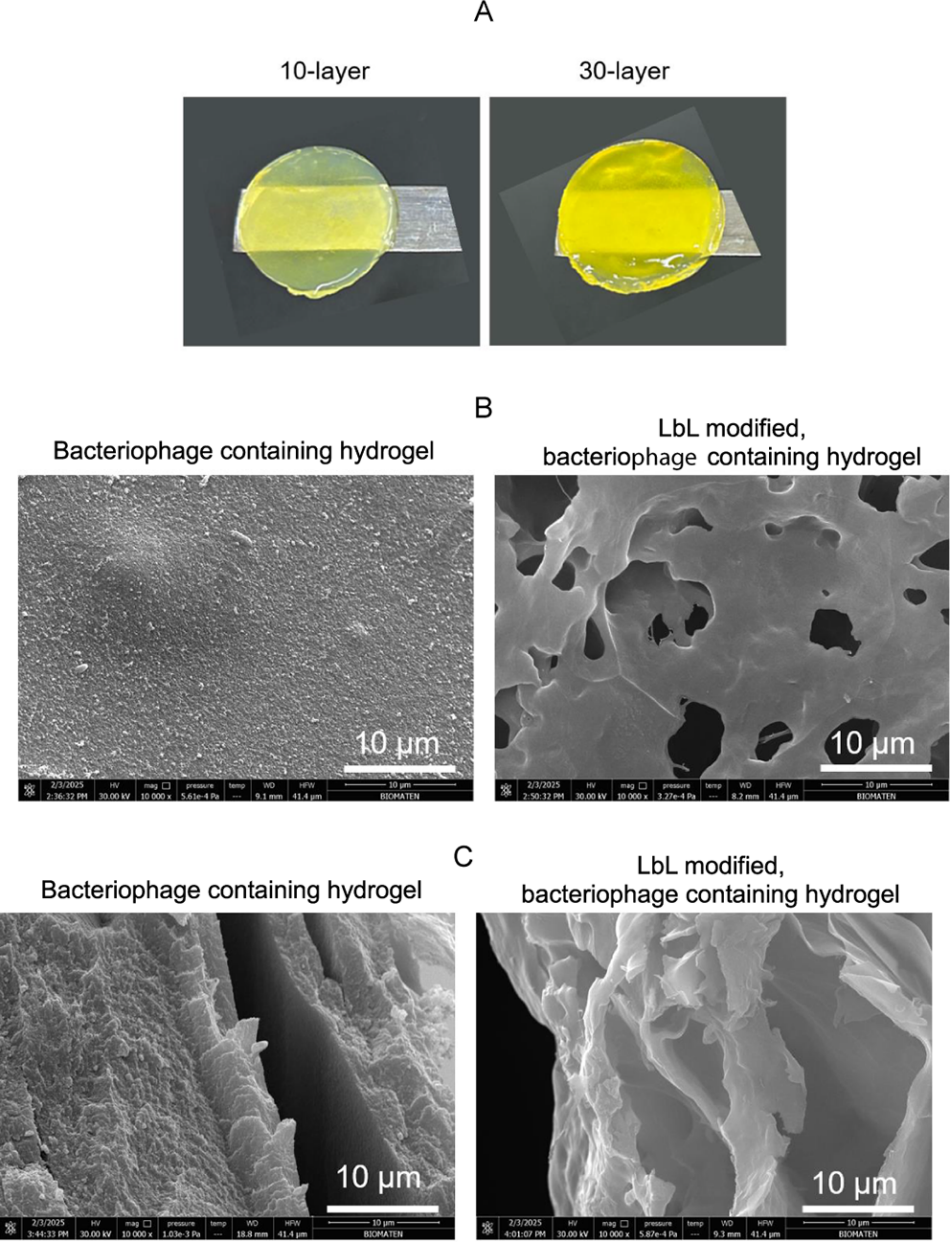
(A) Photographs of alginate hydrogels modified with 10 and 30 layers of PDPA-*b*-βPDMA micelles/alginate. (B) Top-view and (C) cross-sectional SEM images of bacteriophage-containing hydrogels and 30-layer modified, bacteriophage-containing hydrogels.

### 3.3 CUR Release from LbL Modified Hydrogels

Bacteriophage-containing alginate hydrogels were coated with 10 layers of CUR-loaded PDPA-*b*-βPDMA micelles and alginate, and immersed into 10.0 mM phosphate buffer at pH 7.0 and 25 °C; pH 7.0 and 7 °C; pH 5.0 and 25 °C; and pH 5.0 and 7 °C (Figure 6A). For comparison and to understand how LbL films behave on their own, 40 layers of PDPA-*b*-βPDMA micelles/alginate were deposited onto glass slides, and two of the films were immersed into phosphate buffer under the above-mentioned conditions (Figure 6B). As mentioned in Section 3.2.3, polymer building blocks diffuse into the hydrogels during LbL deposition. In order to follow CUR release, it was sufficient to deposit 10 layers on hydrogels, while a film with a similar layer number was not sufficient on glass slides. For this reason, two different glass slides, each coated with 40 layers, were used for the release studies. CUR release was followed using fluorescence spectroscopy by measuring the peak intensity at 535 nm. The amount of CUR released from LbL-modified hydrogels and glass slides was quantified using calibration curves which were prepared under release conditions. Due to the different sizes of the hydrogel and glass slides and the number of layers deposited on them, the concentration of released CUR was normalized separately within each system. In other words, it was aimed to evaluate the effect of pH and temperature on the release rather than the concentration of released CUR. Of note, CUR release could not be determined in PBS at 37 °C due to the instability of the multilayers as well as partial disintegration of the alginate hydrogel. The presence of free polymer chains in the solution made a reliable quantification impossible due to hydrophobic interaction of CUR with polymers affecting the fluorescence intensity measurements.^74^ Both multilayers on glass slides and LbL-modified hydrogels displayed rapid release during the first hour, followed by a more gradual release over a 6-hour period. The only exception to this profile was the pH 5.0/7 °C condition, in which the release was almost complete in the first hour. CUR release at pH 5.0 was greater than that at pH 7.0. This was due to protonation of PDPA core blocks and dissolution of the micelles at pH 5.0, while the release at pH 7.0 was based on self-diffusion of CUR from the micellar cores. Decreasing temperature slightly decreased CUR release from multilayers on glass slides at both pH. However, the effect of decreasing temperature on CUR release from LbL-modified hydrogels was remarkable at pH 5.0, indicating entrapment of CUR in the hydrogel due to enhanced physical interaction between alginate, PDPA-*b*-βPDMA, and CUR as the temperature was decreased.

**Figure 6:**
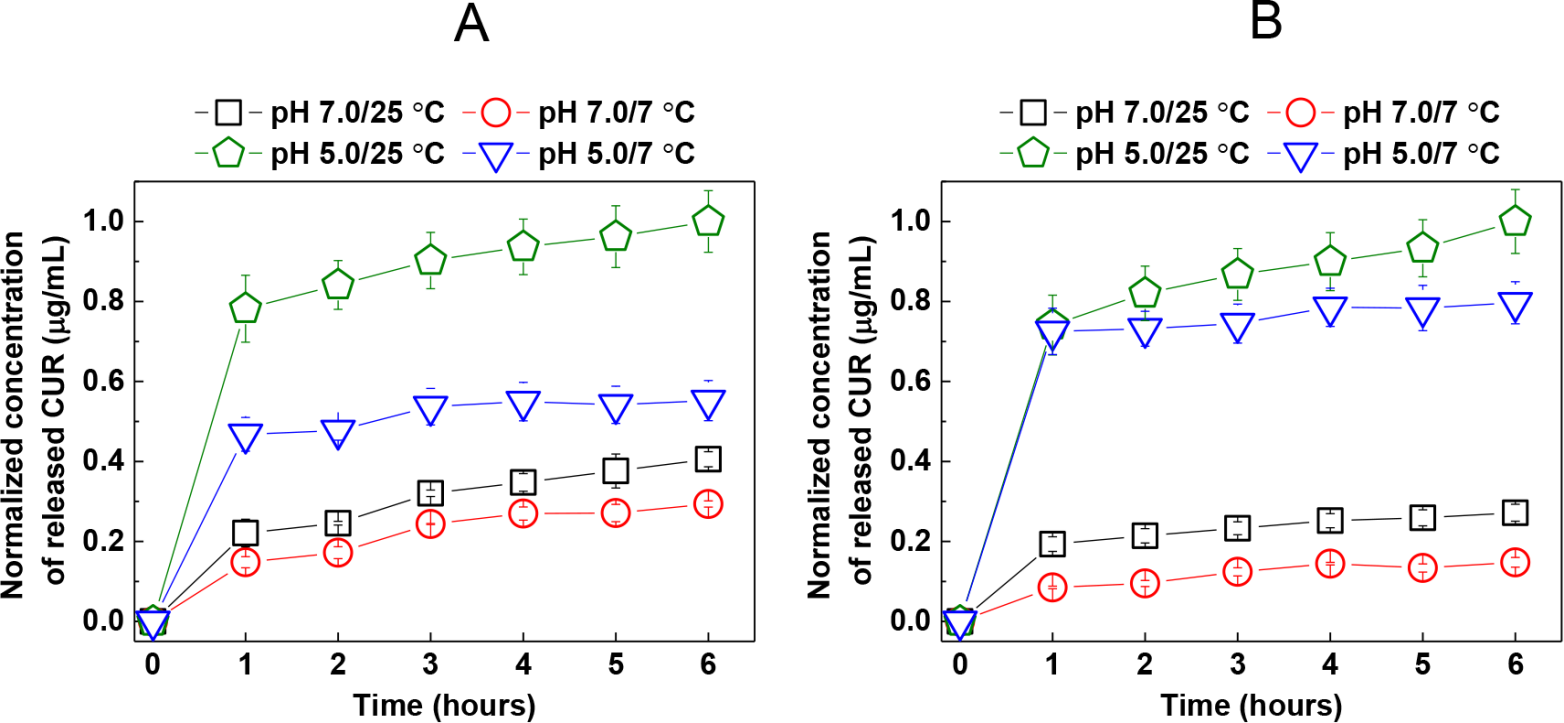
CUR release at pH 7.0/25 °C, pH 7.0/7 °C, pH 5.0/25 °C, and pH 5.0/7 °C from (A) bacteriophage-containing alginate hydrogel modified with 10 layers of CUR-loaded PDPA-*b*-βPDMA micelles/alginate. (B) 40 layers of CUR-loaded PDPA-*b*-βPDMA micelles/alginate, deposited on glass slides.

Our initial hypothesis was that as the temperature exceeded the critical temperature of the βPDMA corona, the chains would transform from extended to globular conformation, forming void-like structures within both multilayer films and hydrogels, and CUR release would be facilitated and increase at 7 °C. However, we found that decreasing the temperature slightly slowed down the release kinetics, and a marked difference in the temperature-dependent release from hydrogels at pH 5.0 was observed. Our results are in agreement with one of our previous studies on PDPA-*b*-βPDMA-containing multilayer films that reported a similar release of pyrene at pH 5.0 for 10 °C and 25 °C but greater for 10 °C as the release medium became more acidic.^75^

In summary, from a chemistry perspective, this part of the study aimed at understanding the effect of UCST-type phase behavior of βPDMA coronal chains on CUR release. Considering the range of applications, given that foods are typically stored and transferred in the cold chain, it is important to study the CUR release under these conditions to ensure the preservation and safety of various food products.

### 3.4 Antibacterial Activity Studies

Kirby–Bauer tests were performed against *Salmonella* Enteritidis at pH 7.0 and pH 5.0 at 37 °C to examine the antibacterial activity of hydrogels as well as the combinatorial effect of bacteriophages and CUR as antibacterial agents. Of note, the antimicrobial effect of CUR against the food-borne pathogen *Salmonella* has been reported.^76^ As shown in Figure 2A, multilayers dissolve almost completely in 30 min after exposure to pH 5.0 at 37 °C. Prior to antibacterial activity tests, long-term stability of multilayers was examined at pH 7.0 at 37 °C. The thickness gradually decreased in the first 6 h and reached 35%. After 24 h, the film loss was 45% (Figure S4). Figure 7 compares the zone diameters obtained with (i) bacteriophage-containing alginate gel, (ii) bacteriophage-containing alginate gel coated with 10 layers of empty PDPA-*b*-βPDMA micelles/alginate, and (iii) bacteriophage-containing alginate gel coated with 10 layers of CUR-loaded PDPA-*b*-βPDMA micelles/alginate. Kirby–Bauer images of the hydrogels are included in Figure S5.

**Figure 7:**
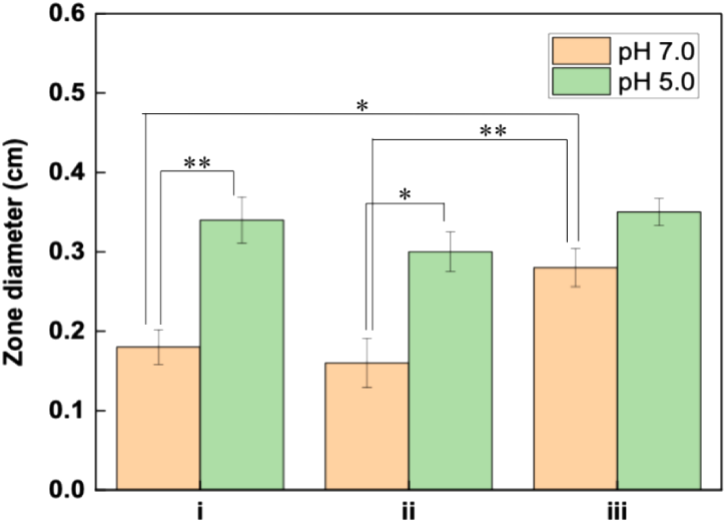
Bar graphs of the net zone diameter of the hydrogels obtained through Kirby–Bauer tests at pH 7.0 and pH 5.0. i: bacteriophage-containing alginate gel, ii: bacteriophage-containing alginate gel coated with 10-layer PDPA-*b*-βPDMA micelles/alginate, iii: bacteriophage-containing alginate gel coated with 10-layer CUR-loaded PDPA-*b*-βPDMA micelles/alginate. Only statistically significant differences are indicated in the figure (* *p*<0.05, ** *p*<0.01); non-significant comparisons are not shown.

The antibacterial activity was greater regardless of the presence of CUR in the hydrogels at pH 5.0. The difference in the zone sizes of the three samples mentioned above was statistically not significant. This result suggests that partial shrinking of the alginate template and expulsion of bacteriophages together with water molecules might be responsible for the enhanced antibacterial activity at pH 5.0. To confirm this speculation, LbL-modified bacteriophage-containing hydrogels were incubated with bacterial cultures at pH 5.0 and pH 7.0. The optical density (OD600) of bacterial cultures was measured after the incubation. The decrease in OD600 values was correlated with greater antibacterial activity and thus greater bacteriophage release from the hydrogels. As seen in Figure S6, OD600 value was remarkably lower for LbL-modified bacteriophage-containing hydrogel at pH 5.0 compared to the bacteria control. On the other hand, OD values of LbL-modified bacteriophage-containing hydrogel and bacteria control were almost similar at pH 7.0. These results support our speculation on greater antibacterial activity due to the greater amount of bacteriophage release from the hydrogels at pH 5.0.

Of note, although the Kirby–Bauer test revealed a clear zone of inhibition at both pH 5.0 and pH 7.0, the bacteriophage release assay did not display a significant difference in bacterial growth at pH 7.0. This discrepancy may be because Kirby–Bauer and the bacteriophage release assay measure different aspects of bacteriophage activity. In the Kirby–Bauer assay, bacteriophage diffusion on agar allows the formation of inhibition zones even with small amounts of bacteriophage release, as the bacteriophages spread and create a visible effect on the solid medium. Therefore, even a limited amount of bacteriophage release from the hydrogel could be enough to form a detectable inhibition zone at pH 7.0. However, in the bacteriophage release assay, bacteriophage activity in liquid media was measured, and the bacteriophages released in small amounts were not enough to show any obvious effect.

It is important to mention that the degradation of CUR accelerates with increasing pH.^77^ It has been reported that the biological activity of CUR decreases once it is oxidized.^78^ Therefore, the contribution of CUR to the antibacterial activity was expected to be lower at pH 7.0. However, contrary to expectations, the combinatorial effect of bacteriophages and CUR was more pronounced at pH 7.0 than at pH 5.0, although the zone diameters were smaller. This result suggests that the combinatorial effect of CUR could only be observed at a lower amount of bacteriophage release. This result is in good agreement with one of our recent studies, which reported that the combinatorial effect of CIP and CUR was lost at increasing CIP concentrations.^25^ Note that that bare alginate hydrogels (no bacteriophages) coated with 10 layers of CUR-loaded PDPA-*b*-βPDMA micelles and alginate did not lead to zone formation (Figure S7), suggesting that the amount of CUR released from the hydrogel was not enough to show antibacterial activity alone but contributed to an enhanced antibacterial effect when used together with bacteriophages.

As mentioned in Section 3.2.2, the concentration of CUR in the micelle solution had to be significantly lower than the MIC value of CUR against *Salmonella*Enteritidis (132.5 μg/mL)^79^ because more ethanol had to be added to the polymer solution to increase the concentration of CUR, which posed a problem for the solubility of the polymer. This is probably the reason we did not observe antibacterial activity with bacteriophage-free hydrogels that were LbL-modified using CUR-loaded PDPA-*b*-βPDMA micelles. For this reason, we investigated the potential enhancing effect of CUR when combined with bacteriophages. The antimicrobial effect of CUR might be due to different mechanisms, including disruption of bacterial membranes, inhibition of biofilm formation, induction of oxidative stress, and interference with bacterial cell division.^80^ Among these mechanisms, the disruption of the bacterial membrane is particularly significant, as CUR’s hydrophobic nature allows it to integrate into bacterial membranes, which increases their permeability. This increasing permeability facilitates the injection of bacteriophage genetic material, thereby enhancing bacteriophage replication efficiency and overall antibacterial efficacy. Supporting this approach, recent studies have shown that CUR can still contribute to antibacterial activity below the MIC concentrations when combined with other agents and also bacteriophages. For example, in a study by Karajeh et al.,^81^ CUR at sub-MIC levels exhibited synergistic antibacterial effects when combined with meloxicam and diclofenac, resulting in enhanced inhibition of *Staphylococcus aureus* and *Enterococcus faecium*. In a recent study by Janesomboon et al.,^52^ a synergistic effect was observed when CUR was used in combination with bacteriophage at a CUR concentration of 400 μg/mL, which is below the MIC value against *Acinetobacter baumannii*, as the MIC values for this pathogen range between 0.625 and 2.5 mg/mL.^82^ They reported that the combination had reduced bacterial concentration to undetectable levels within 1 h. In addition, if the concentrations of both phage and CUR were doubled, the effectiveness of the combination lasted for up to 5 h.^52^

In summary, these results showed us that the bacteriophages in the hydrogel were indeed mainly responsible for the antibacterial activity, and CUR acted in a combinatorial manner to enhance the antibacterial activity at pH 7.0. Of note, hydrogels that were used as negative controls did not provide zone formation as expected. Scheme 2 represents bacteriophage and CUR release from LbL-coated hydrogel under pH 7.0/37 °C and pH 5.0/37 °C conditions.

**Scheme 2:**
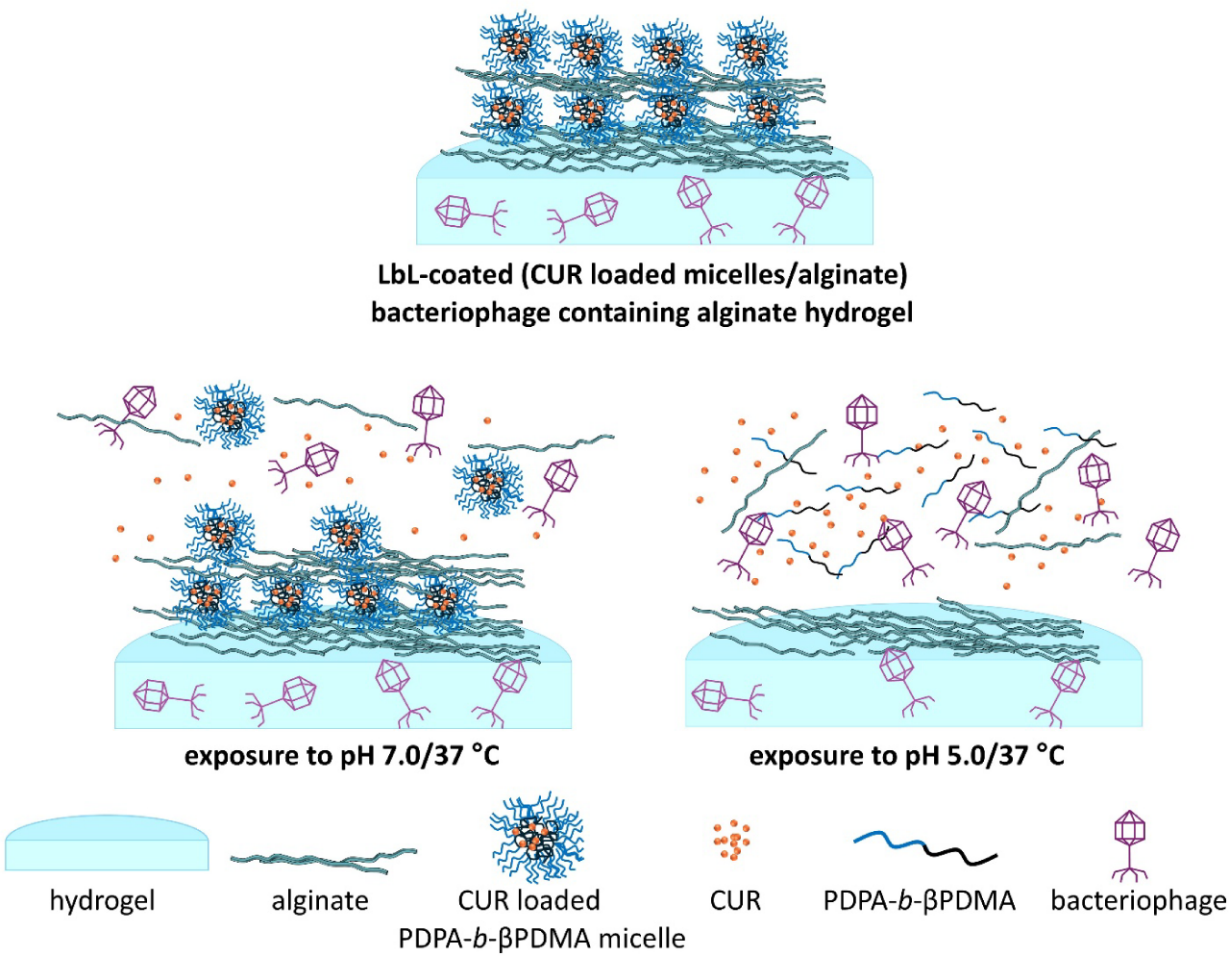
Schematic Representation of LbL-Coated (CUR-Loaded PDPA-*b*-βPDMA Micelles/Alginate Deposition) Bacteriophage-Containing Alginate Hydrogel before and after Exposure to pH 7.0/37 °C and pH 5.0/37 °C

## 4 Conclusion

This study first aimed at the preparation and characterization of multilayers with UCST-type phase behavior using zwitterionic PDPA-*b*-βPDMA micelles and alginate. We observed that approaching the self-assembly temperature to the critical point of zwitterionic coronal chains resulted in thicker films but had no effect on the stability of multilayers against pH change. On the other hand, the post-assembly temperature was critical to the stability of LbL films. The stability increased as the post-assembly temperature decreased from 37 °C to 7 °C. These multilayers could then be successfully employed on alginate hydrogels to produce a dual antibacterial agent-releasing platform. Prior to LbL assembly, CUR was loaded into PDPA-*b*-βPDMA micelles, and *Salmonella* bacteriophages were embedded in the alginate hydrogel. CUR release from LbL-modified hydrogels was greater at pH 5.0 than at pH 7.0 due to the dissolution of the micellar cores. Decreasing the temperature did not make a significant impact on the amount of CUR released at pH 7.0 when the hydrogels were in the swollen state. On the other hand, decreasing the temperature reduced CUR release at pH 5.0 when the hydrogels shrank, possibly due to the entrapment of hydrophobic CUR in the hydrogels as the association among alginate chains enhanced.

The antibacterial activity was greater at pH 5.0 regardless of whether CUR was loaded into micelles, suggesting that bacteriophages were mainly responsible for the antibacterial activity. The greater antibacterial activity at pH 5.0 can be explained by the greater amount of bacteriophage release due to partial shrinking of the alginate hydrogel, providing expulsion of bacteriophages together with water molecules. CUR enhanced the antibacterial activity provided by bacteriophages at pH 7.0, indicating that the bacteriophage and CUR acted in a combinatorial manner. This effect was statistically not significant at pH 5.0, indicating that the contribution of CUR could be more pronounced at lower amounts of bacteriophage release from the hydrogels.

Overall, this study generated fundamental information toward surface modification of bacteriophage-containing alginate hydrogels through the LbL self-assembly technique for antibacterial applications.

## Supplementary Material



## Notes

The GPC chromatogram of PDPA-*b*-PDMA diblock copolymer; proton NMR spectra of precursor PDPA-*b*-PDMA diblock copolymer and selectively betainized PDPA-*b*-βPDMA diblock copolymer; number-average hydrodynamic size distribution and zeta-potential distribution of PDPA-*b*-βPDMA micelles at pH 7.0 and at 25 °C; TEM image of PDPA-*b*-βPDMA micelles at pH 7.0; hydrodynamic size distribution of bacteriophages by intensity at pH 7.0 and TEM image of bacteriophages; stability of 12-layer PDPA-*b*-βPDMA micelles/alginate film (prepared at pH 7.0/25 °C) in 10 mM phosphate buffer solution at pH 7.0/37 °C; Kirby–Bauer images of the alginate hydrogels at pH 5.0 and pH 7.0 and at 37 °C; inhibition of bacterial growth in liquid culture by the bacteriophage containing hydrogel at pH 5.0 and pH 7.0 and at 37 °C; Kirby–Bauer images of the bare alginate hydrogels (no bacteriophages) at pH 7.0/37 °C (PDF)
