# Supplementary material for: Layer-by-Layer Coated Alginate Hydrogels with Antibacterial Activity Based on Bacteriophage and Curcumin Combination
Source: ACS Omega. 2025 Jul 23;10(30):33108–23. doi: 10.1021/acsomega.5c02562 (PMC12332794; doi:10.1021/acsomega.5c02562)
Supplement: Supplementary file 1 [file ao5c02562_si_001.pdf]

Supporting Information

Layer-by-layer Coated Alginate Hydrogels with Antibacterial Activity Based on Bacteriophage and Curcumin Combination

Ayse Guren[a], Aysenur Yucefaydali[b], Dilara Gundogdu[c], Artun Bozkurt[c], Vural Butun[d], Yesim Soyer[a,b], Irem Erel-Goktepe [a,c,e,*]

[a] *Department of Biochemistry, Middle East Technical University, 06800 Cankaya, Ankara, Türkiye*

[b] *Department of Food Engineering, Middle East Technical University, 06800 Cankaya, Ankara, Türkiye*

[c] *Department of Chemistry, Middle East Technical University, 06800 Cankaya, Ankara, Türkiye*

[d] *Department of Chemistry, Eskisehir Osmangazi University, 26480, Eskisehir, Türkiye*

[e] *Center of Excellence in Biomaterials and Tissue Eng. Middle East Technical University, 06800, Cankaya, Ankara, Türkiye*

Abbreviations: Poly[2-(diisopropylamino)ethyl methacrylate]-*block*-poly[3-dimethyl(methacryloyloxyethyl)ammonium propanesulfonate] (PDPA-b-βPDMA), Curcumin (CUR), layer-by-layer (LbL), upper critical solution temperature (UCST).

*To whom correspondence should be addressed. Telephone: +90 312 210 3233. E-mail:erel@metu.edu.tr

A

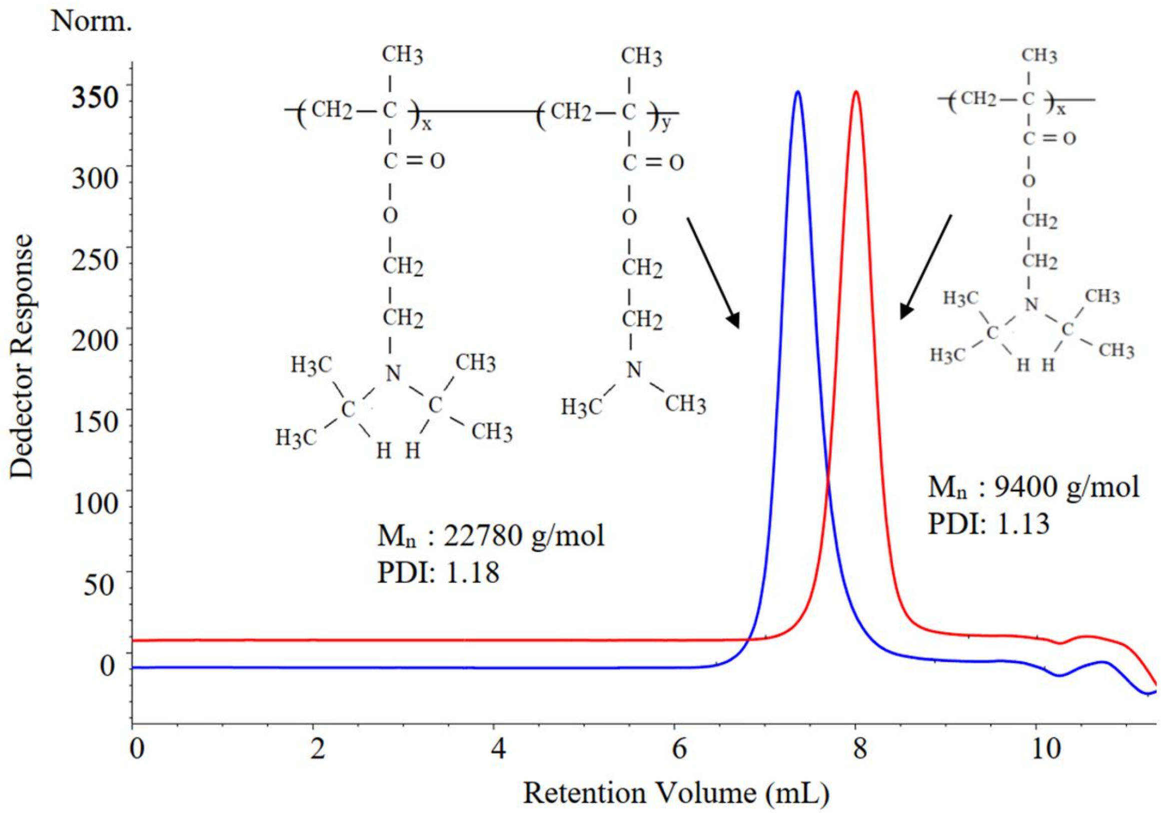

Norm.
Dedector Response
350
300
250
200
150
100
50
0
0
2
4
6
8
10
Retention Volume (mL)
Mn : 22780 g/mol
PDI: 1.18
Mn : 9400 g/mol
PDI: 1.13

B

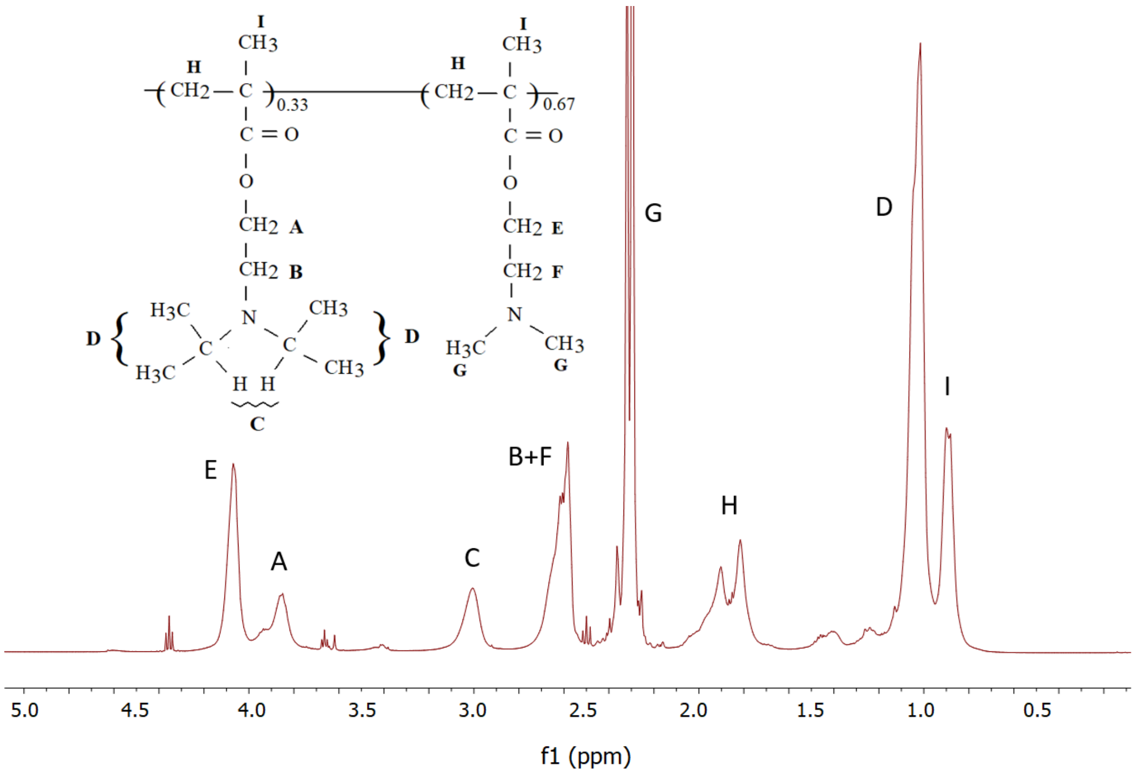
I
CH3
H
CH2
0.33
C = O
O
CH2 A
CH2 B
N
H3C
CH3
D
H3C
CH3
D
H H
C
I
CH3
H
CH2
0.67
C = O
O
CH2 E
CH2 F
N
H3C
G
CH3
G
G
D
I
E
A
C
B+F
H
5.0
4.5
4.0
3.5
3.0
2.5
2.0
1.5
1.0
0.5
f1 (ppm)

C

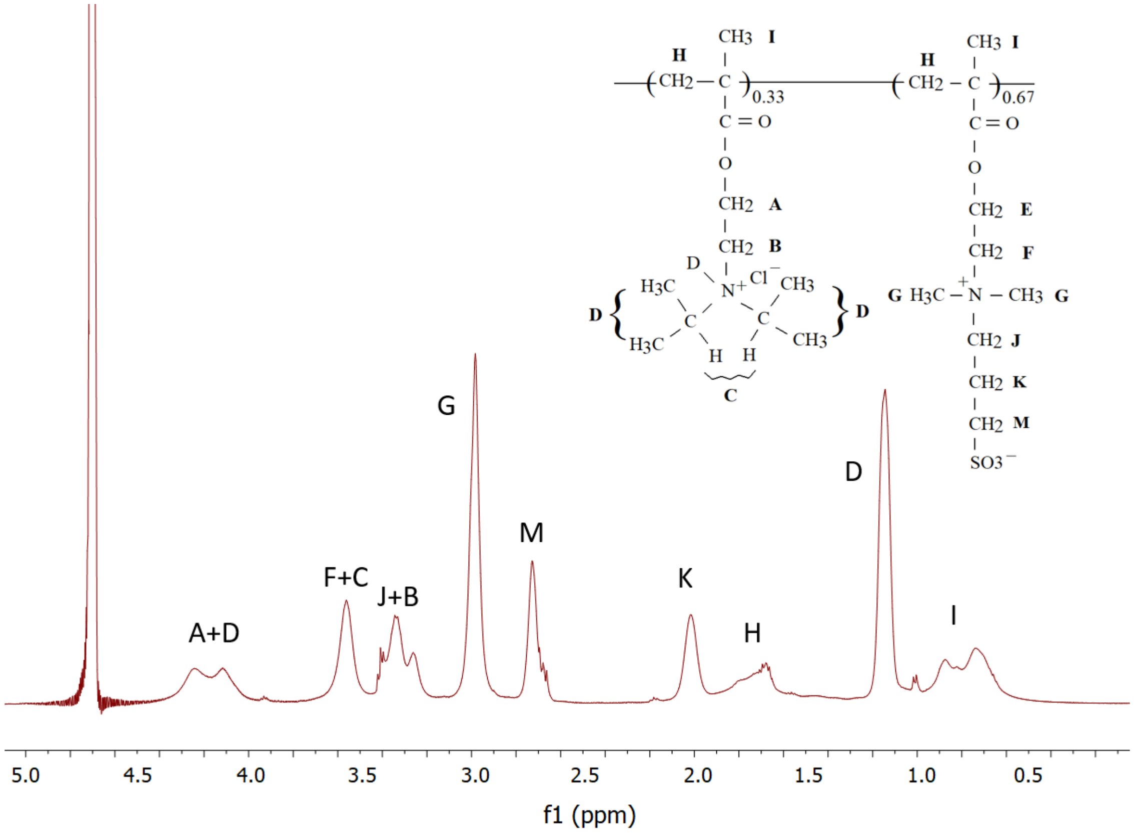

Figure S1. (A) GPC chromatogram of PDPA-*b*-PDMA diblock copolymer. Proton NMR spectra of precursor PDPA-*b*-PDMA diblock copolymer in $CDCl_3$ **(B)** and selectively betainized PDPA-*b*-βPDMA diblock copolymer in D_2O/DCl at pH 2.0 **(C)**.

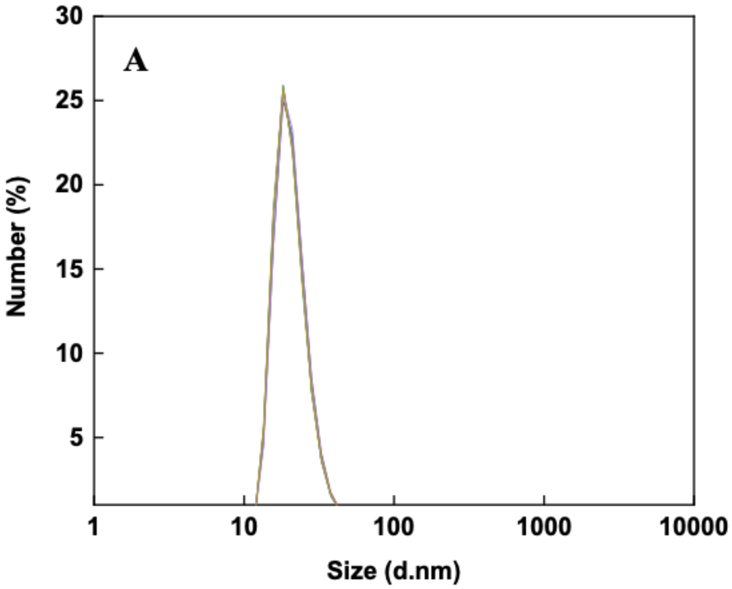
A
Number (%)
30
25
20
15
10
5
1
10
100
1000
10000
Size (d.nm)

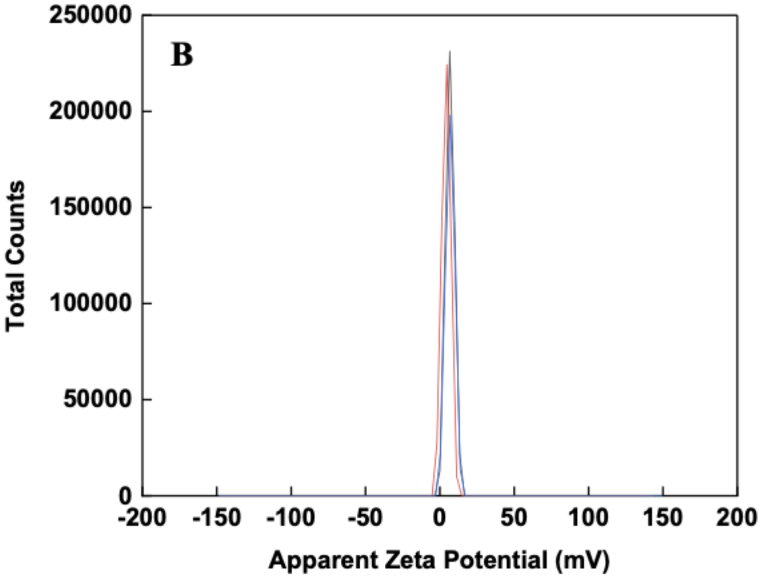
B
Total Counts
250000
200000
150000
100000
50000
0
-200
-150
-100
-50
0
50
100
150
200
Apparent Zeta Potential (mV)

C

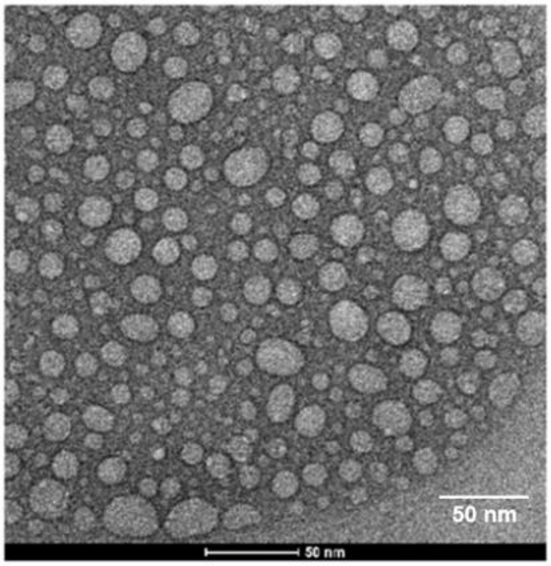

Figure S2. (A) Number average hydrodynamic size distributions of PDPA-*b*-βPDMA micelles at pH 7.0 and at 25 ℃. **(B)** Zeta-potential distribution of βPDMA-*b*-PDPA micelles at pH 7.0 and at 25 ℃. Size distribution curves obtained from several individual measurements of the same sample are represented with different colors. **(C)** TEM image of PDPA-*b*-βPDMA micelles at pH 7.0.

A

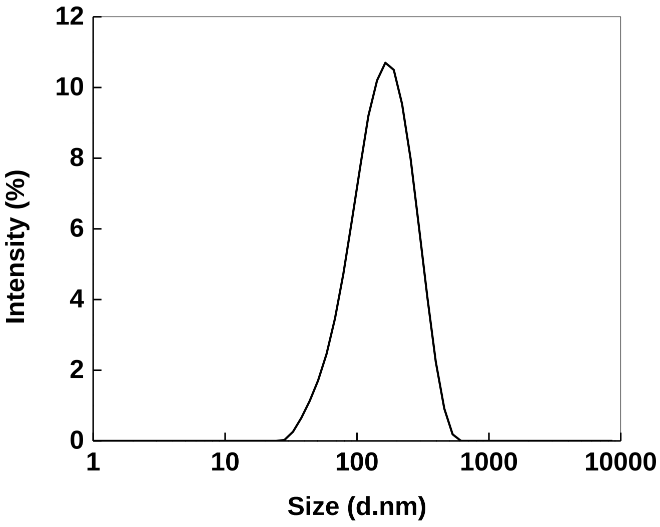

B

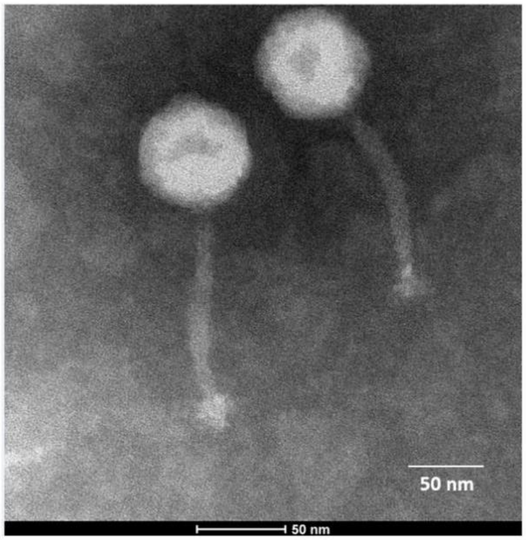

Figure S3. (A) The hydrodynamic size distribution of bacteriophages by intensity at pH 7.0. **(B)** TEM image of bacteriophages.

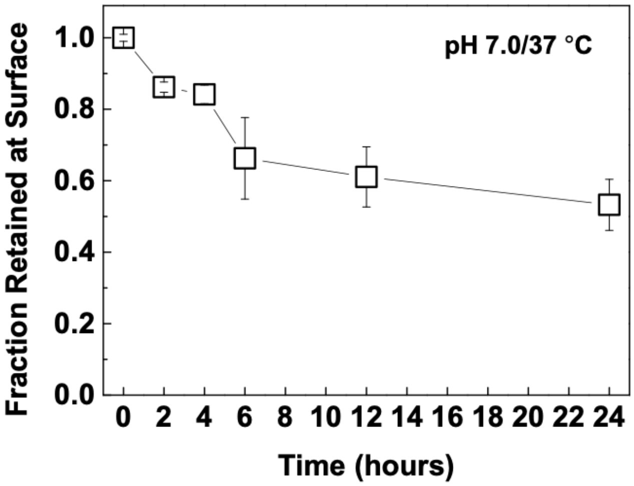

Figure S4. Fraction retained at the surface of 12-layer PDPA-*b*-βPDMA micelles/alginate film (prepared at pH 7.0/25 ℃) as a function of time after exposure to 10 mM phosphate buffer solution at pH 7.0/37 ℃. The fractions were calculated by dividing the film thickness at a specific time by the initial thickness.

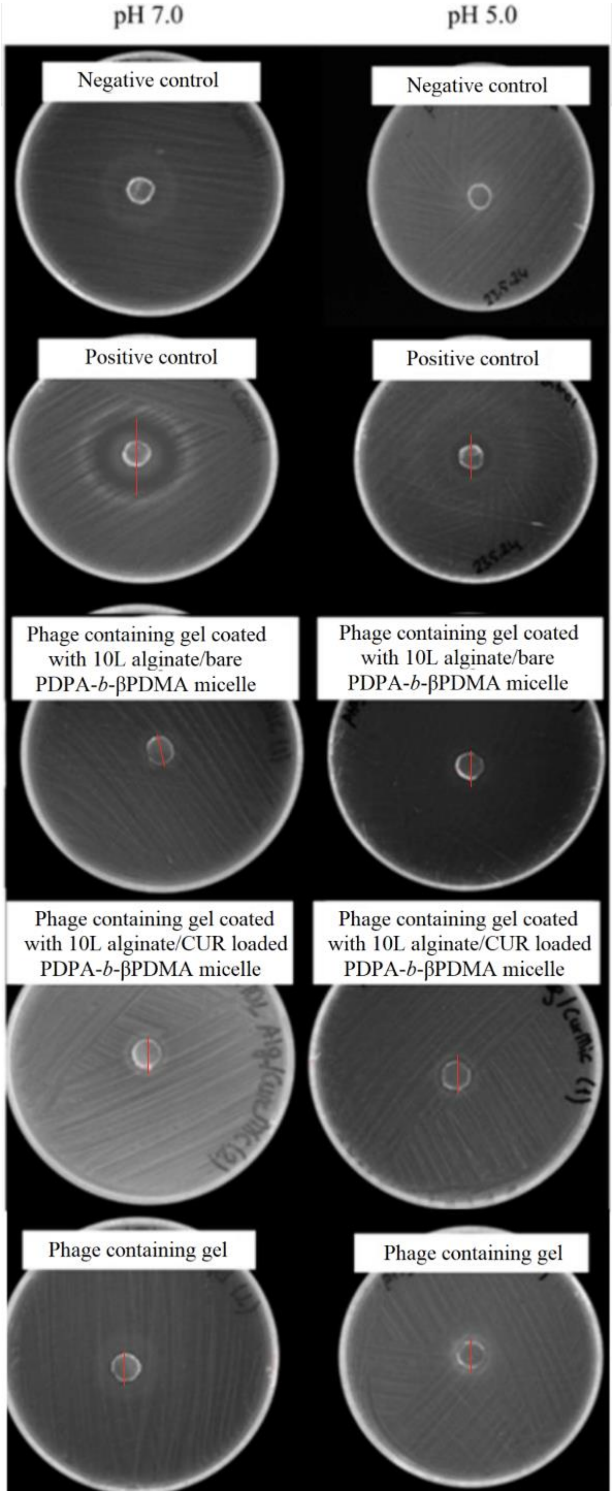

Figure S5. Kirby Bauer images of the alginate hydrogels at pH 7.0 and pH 5.0 and at 37 ℃.

A

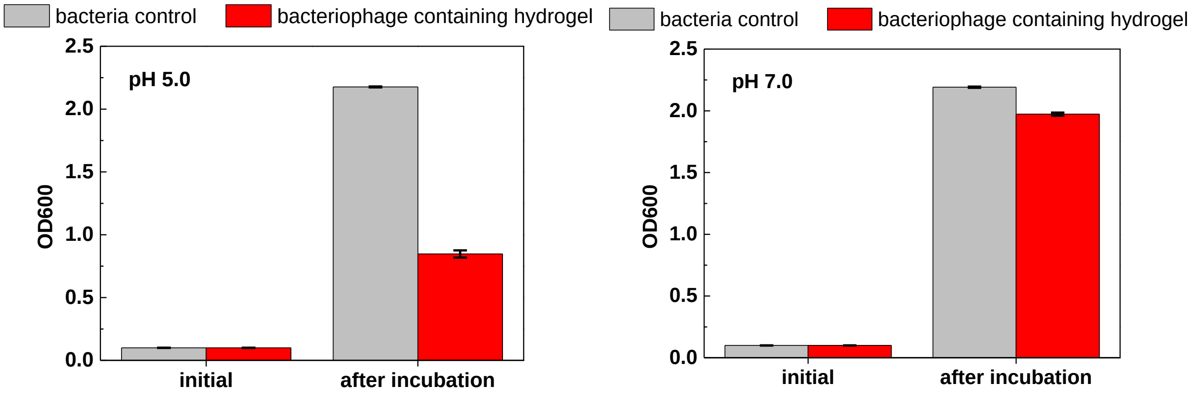

B

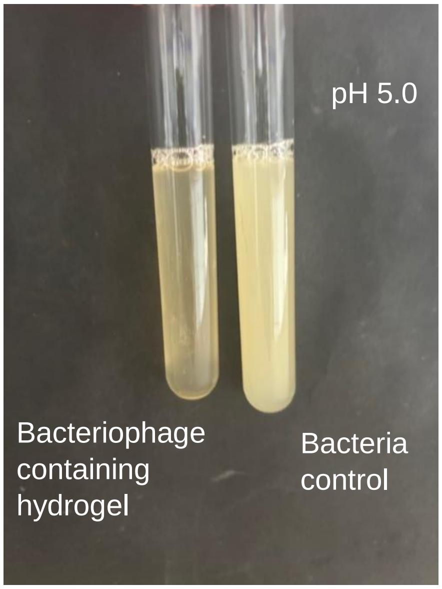

Figure S6. **(A)** Inhibition of bacterial growth in liquid culture by the bacteriophage-loaded hydrogel coated with 10-layer PDPA-*b*-βPDMA Micelle/Alginate compared to the control group without the hydrogel at pH 5.0 and pH 7.0 **(B)** LB broth containing only bacteria (control) (right) and both bacteria and LbL coated bacteriophage containing hydrogel (left) at pH 5.0.

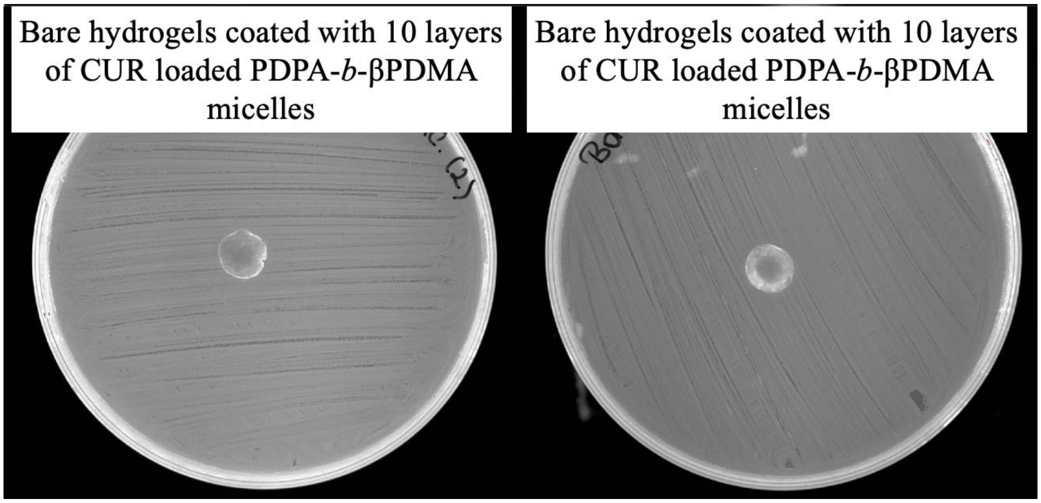

Figure S7. Kirby Bauer images of the bare alginate hydrogels (no bacteriophages) coated with 10 layers of CUR loaded PDPA-*b*-βPDMA micelles and alginate.
